# Supplementary material for: Quantification of cell-free DNAfor the analysis of CD19-CAR-T cells during lymphoma treatment
Source: Mol Ther Methods Clin Dev. 2021 Oct 28;23:539–50. doi: 10.1016/j.omtm.2021.10.009 (PMC8606297; doi:10.1016/j.omtm.2021.10.009)
Supplement: Document S1. Figures S1–S9 [file mmc1.pdf]

OMTM, Volume *23*

Supplemental information

Quantification of cell-free DNA for the analysis of CD19-CAR-T cells during lymphoma treatment

Thomas Mika, Julia Thomson, Verena Nilius-Eliliwi, Deepak Vangala, Alexander Baraniskin, Gerald Wulf, Susanne Klein-Scory, and Roland Schroers

Supplemental Material

Supp. Fig. 1

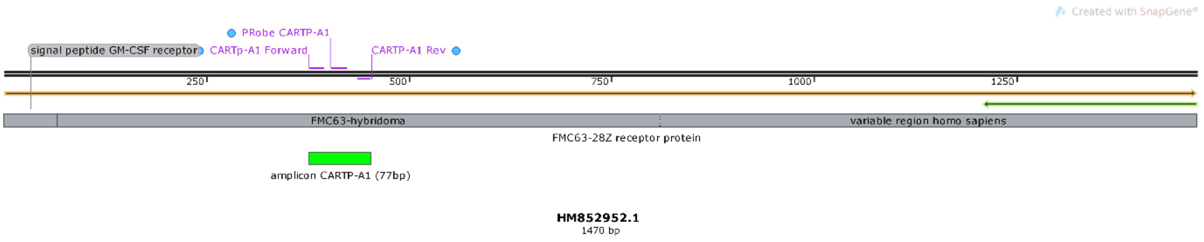

***Supplement Figure 1*: Primer-probe-pair and amplicon within the CD19-CAR gene**

The primer-probe pair to detect cfCAR-DNA was designed to detect an amplicon of 77 bp in length. Reference primer-probe pair was used as previously published [10].

Supp. Fig. 2

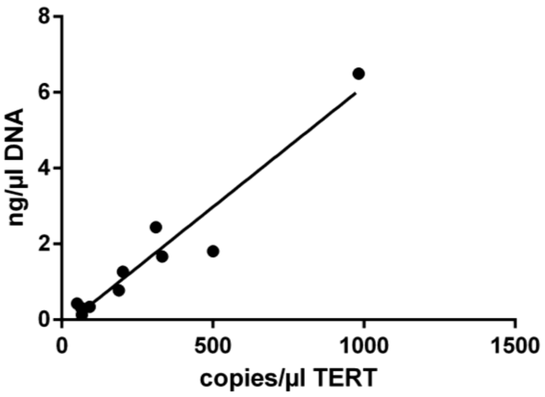

Supplement Figure 2:* Correlation of cfDNA and copies/µl *TERT

Correlation of patients' cfDNA (n=10), isolated from plasma samples, and *TERT*. The amount of cfDNA in the analyzed eluate correlated well with the measured quantity of *TERT* (copies/µl eluate) assessed by ddPCR ($r^2 = 0.93$, $p<0.001$).

Supp. Fig. 3

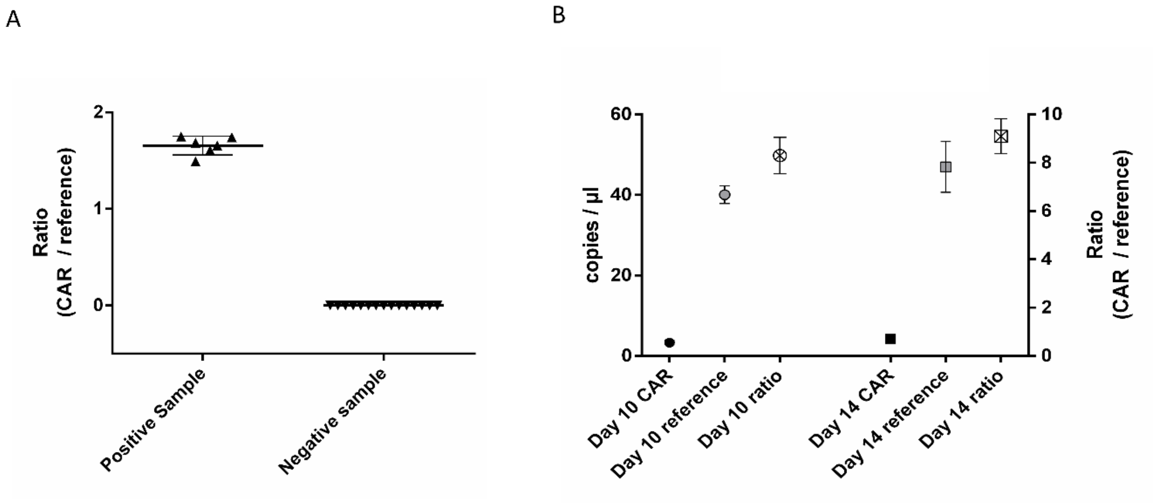

***Supplement Figure 3*: Analytical Performance of the modified ddPCR-assay**

A: Repeated measurements of positive (n = 6) and negative control samples (n = 15) confirmed high reproducibility and precision of the modified ddPCR assay (SD 0.097 and 0.001). Limit of blank was 0.07 copies/µl. For subsequent analyses results below 0.07 copies/µl were set to 0.

B: The reproducibility of the complete workflow of the new ddPCR assay, encompassing DNA isolation from plasma and subsequent PCR amplification in repeated measurements of two blood samples collected from patients treated with axi-cel, was tested. In brief, DNA was isolated from the two samples 4- and 3-times, respectively. High reproducibility of the assay with a standard deviation of the ratio (CAR / reference) of 0.75 and 0.72 was seen in the two experiments.

Supp. Fig. 4

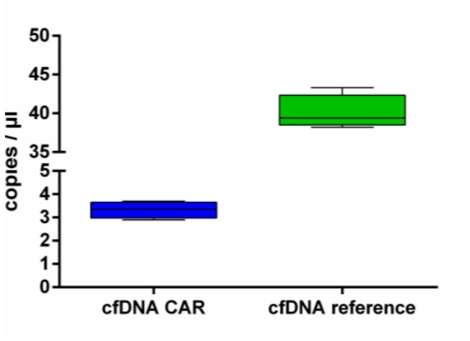

Supplement Figure 4: **Repetitive DNA-isolation from a blood sample 10 days after axi-cel infusion.**

A blood sample from one patient after axi-cel infusion was processed 4-times to obtain cfDNA. Mean copies/µl cfDNA CAR was 3.325 (±0.35). Mean copies/µl reference cfDNA was 40.08 (±2.223).

Supp. Fig. 5

A

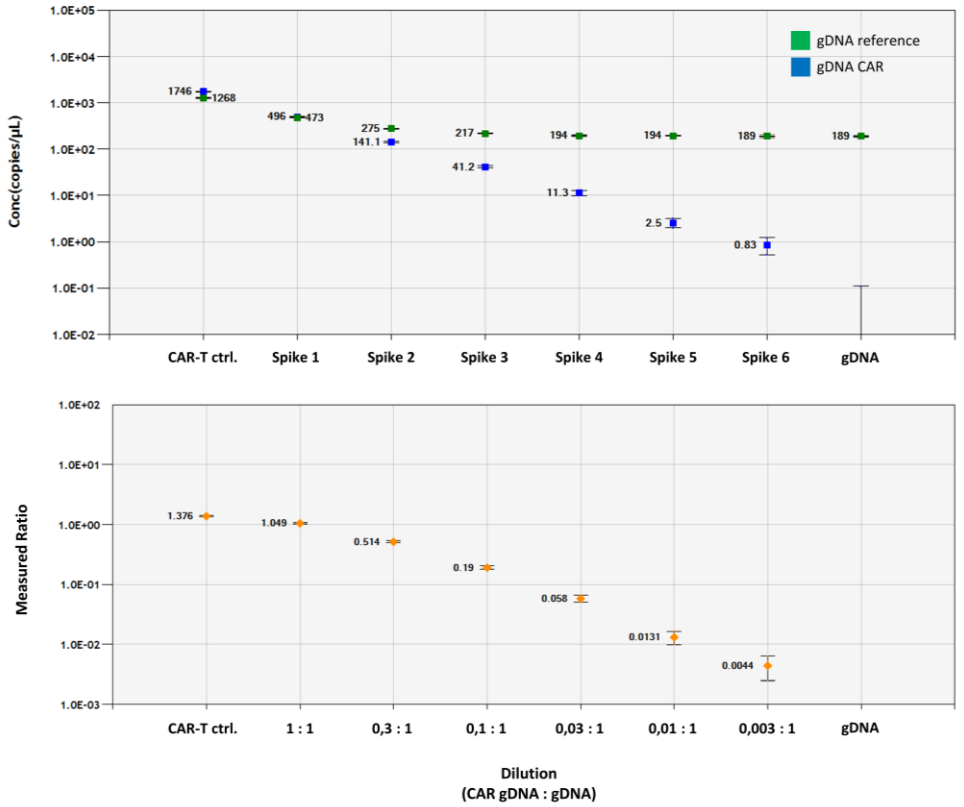
gDNA reference
gDNA CAR
Conc(copies/µL)
1.0E+05
1.0E+04
1.0E+03
1.0E+02
1.0E+01
1.0E+00
1.0E-01
1.0E-02
1746
1268
496
473
275
141.1
217
41.2
194
11.3
194
2.5
189
0.83
189
CAR-T ctrl.
Spike 1
Spike 2
Spike 3
Spike 4
Spike 5
Spike 6
gDNA
Measured Ratio
1.0E+02
1.0E+01
1.0E+00
1.0E-01
1.0E-02
1.0E-03
1.376
1.049
0.514
0.19
0.058
0.0131
0.0044
CAR-T ctrl.
1 : 1
0,3 : 1
0,1 : 1
0,03 : 1
0,01 : 1
0,003 : 1
gDNA
Dilution
(CAR gDNA : gDNA)

B

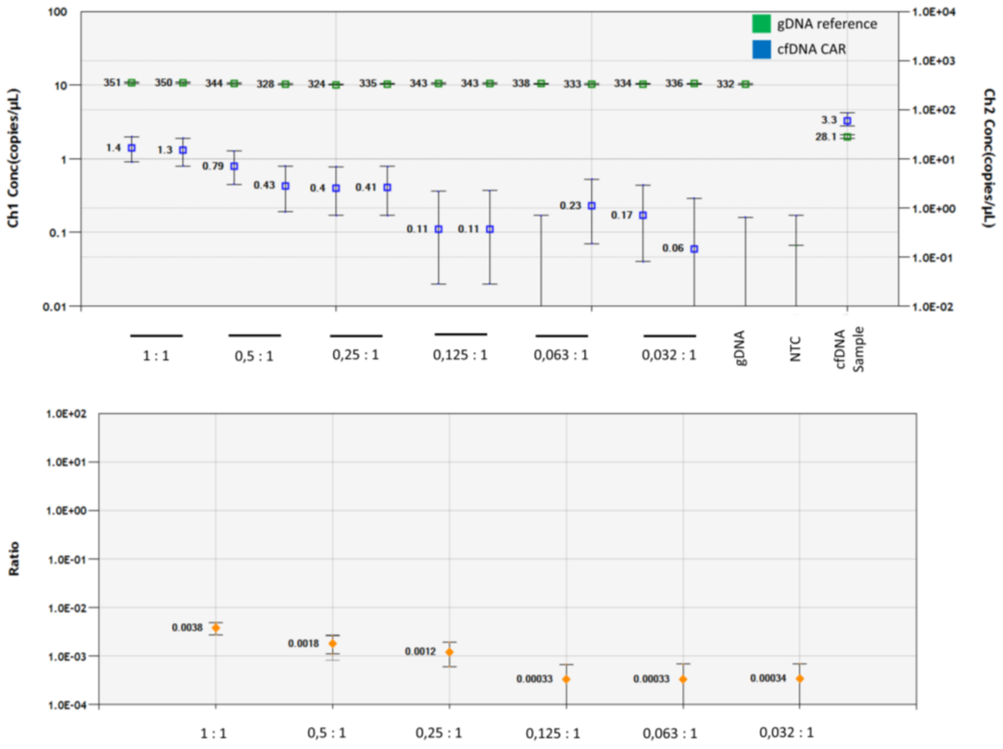
gDNA reference
cfDNA CAR
Ch1 Conc(copies/µL)
Ch2 Conc(copies/µL)
100
10
1
0.1
0.01
1.0E+04
1.0E+03
1.0E+02
1.0E+01
1.0E+00
1.0E-01
1.0E-02
351
350
344
328
324
335
343
343
338
333
334
336
332
1.4
1.3
0.79
0.43
0.4
0.41
0.11
0.11
0.23
0.17
0.06
3.3
28.1
1 : 1
0,5 : 1
0,25 : 1
0,125 : 1
0,063 : 1
0,032 : 1
gDNA
NTC
cfDNA Sample
Ratio
1.0E+02
1.0E+01
1.0E+00
1.0E-01
1.0E-02
1.0E-03
1.0E-04
0.0038
0.0018
0.0012
0.00033
0.00033
0.00034
1 : 1
0,5 : 1
0,25 : 1
0,125 : 1
0,063 : 1
0,032 : 1

Supplement Figure 5: **Limit of Detection of the modified ddPCR-assay**

A: Dilution of CAR gDNA in reference gDNA. For every sample, CAR gDNA (spike1-6) was diluted in a constant amount of reference gDNA (30 ng). At least 0,01 ng CAR gDNA was reliably detectable in a background of 30 ng reference gDNA (0.03%).

B: Dilution of patient derived cfDNA in reference gDNA. For each sample, cfDNA from a patient derived plasma sample was diluted in 40 ng of reference gDNA. cfDNA was reliably detectable in a ratio of 0.1% and still detectable at lower concentrations. Based on the assumption that 308 copies were evident in the cfDNA sample (3.3 cop/µl * (20/5)*(70/3) = 308 copies / ml plasma), limit of detection was between 38.5-77 copies/ml Plasma (0.125 and 0.25 dilution factors).

Supp. Fig. 6

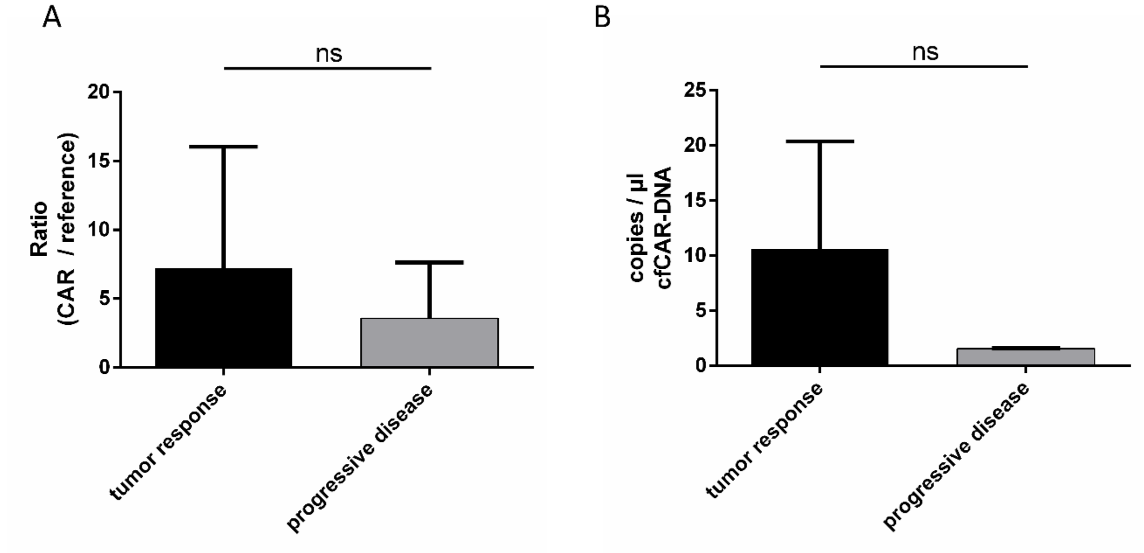

***Supplement Figure 6*: The ratio's peak and the cfCAR-DNAs' peak of patients with tumor response and disease progression.**

A: The ratio's peak of cfCAR-DNA to reference cfDNA tended to be higher in patients responding upon axi-cel treatment. The difference is not significant (p = 0.334). Tumor response: n = 8, progressive disease: n = 4.

B: Maximum absolute amount (copies/µl) of cfCAR-DNA is lower in patients not responding. Difference is not significant (p = 0.151). Tumor response: n = 8, progressive disease: n = 4.

Supp. Fig. 7

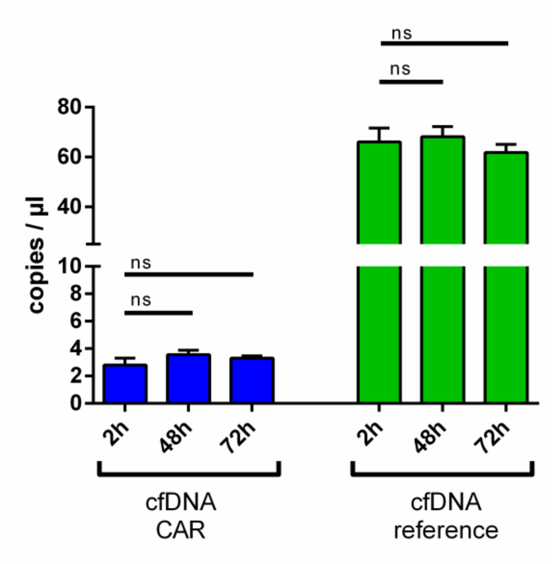

Supplement Figure 7: **Analysis of cfDNA in a patient 14 days after axi-cel infusion.** Three blood samples from one patient at one time point were obtained and further processed after 2 h, 48 h, and 72 h. Each sample was analyzed three times. Absolute copies/µl of cfCAR-DNA and reference cfDNA were constant over time. (cfCAR-DNA; $p = 0.089$ and $p = 0.177$, cfDNA reference; $p = 0.622$ and $p = 0.311$).

Supp. Fig. 8

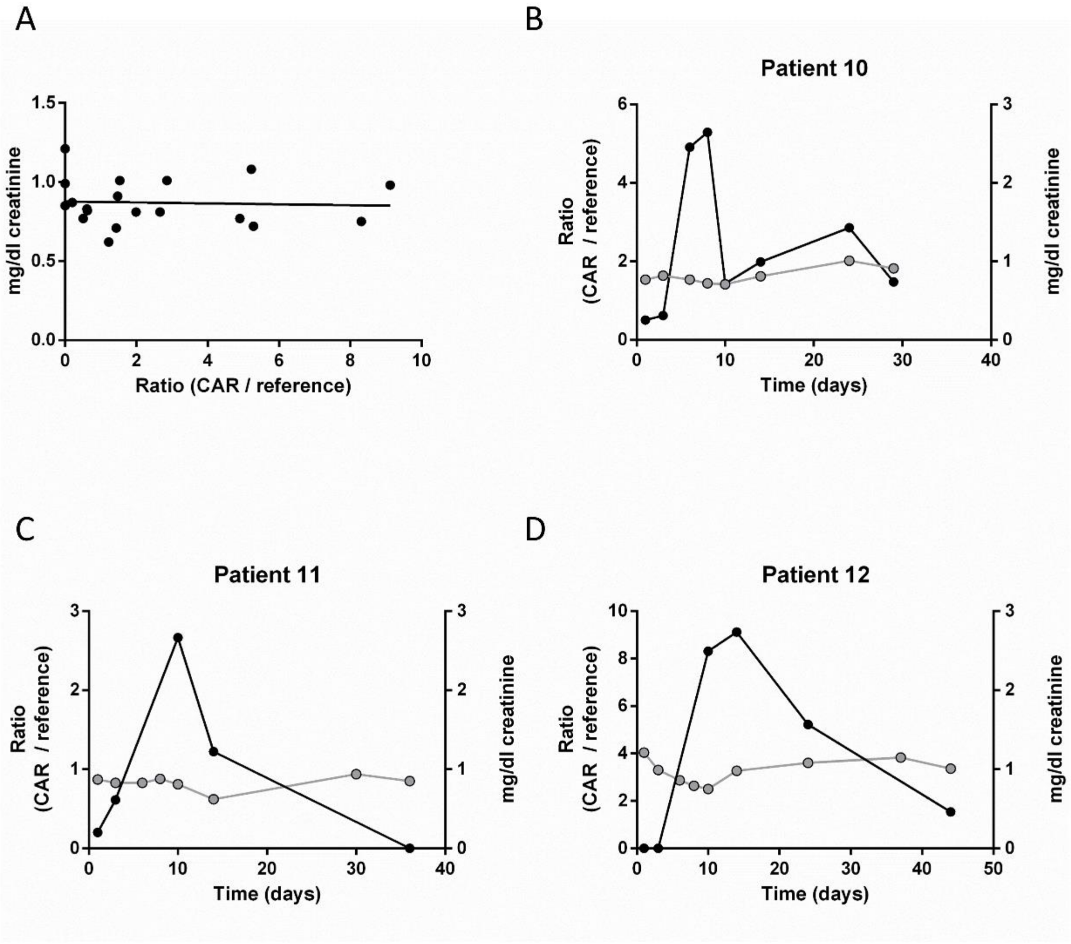

Supplement Figure 8: **Correlation of cfDNA and renal function**

A: Pearson correlation of serum creatinine levels and the ratio of the cfCAR-DNA (r^2 = 0.003).

B-D: The ratio of cfCAR-DNA to reference cfDNA and serum creatinine levels of patients treated with axi-cel over time. Black dots: ratio, grey dots: mg/dl creatinine.

Supp. Fig. 9

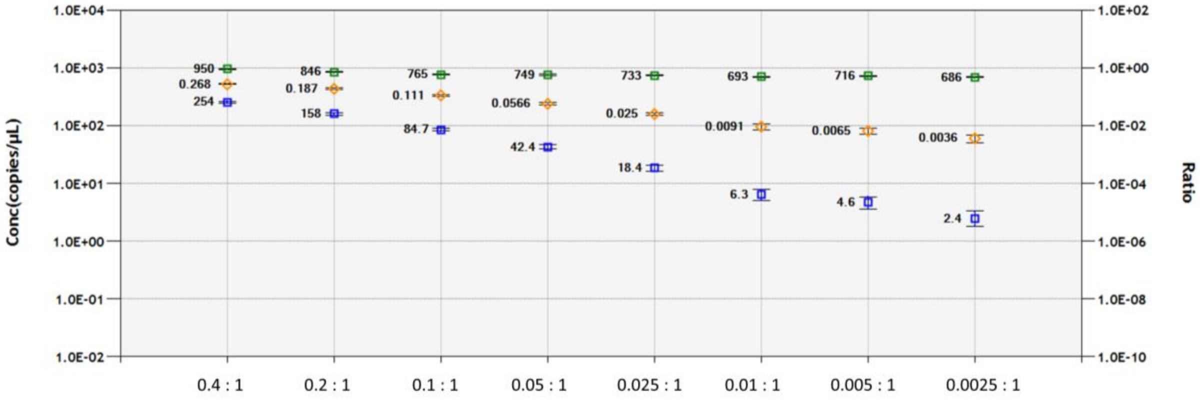

Supplement Figure 9**: Dilution of Karpas derived gDNA in reference gDNA**

Karpas gDNA, comprising IgH-BCL2 translocation, was spiked into 50 ng of reference human gDNA. Subsequently, IgH-BCL2 copies were assessed by an assay previously developed by Drandi et al. [37]. IgH-BCL2 copies were reliably detectable in a ratio of 0.0025 (2.5×10^{-3}).
