# Supplementary material for: Quantification of cell-free DNAfor the analysis of CD19-CAR-T cells during lymphoma treatment
Source: Mol Ther Methods Clin Dev. 2021 Oct 28;23:539–50. doi: 10.1016/j.omtm.2021.10.009 (PMC8606297; doi:10.1016/j.omtm.2021.10.009)
Supplement: Document S2. Article plus supplemental information [file mmc2.pdf]

Molecular Therapy
Methods & Clinical Development
Original Article

Quantification of cell-free DNA for the analysis of CD19-CAR-T cells during lymphoma treatment

Thomas Mika,[1] Julia Thomson,[2] Verena Nilius-Eliliwi,[1] Deepak Vangala,[1] Alexander Baraniskin,[1,3] Gerald Wulf,[2] Susanne Klein-Scory,[1,4] and Roland Schroers[1]

[1]Department of Medicine, Hematology and Oncology, Ruhr University Bochum, 44892 Bochum, Germany; [2]Clinic for Hematology and Medical Oncology, Georg-August University, 37075 Göttingen, Germany; [3]Department of Hematology, Oncology and Palliative Care, Evangelisches Krankenhaus Hamm, 59063 Hamm, Germany; [4]IMBL, Universitätsklinikum Knappschaftskrankenhaus Bochum, 44892 Bochum, Germany

Chimeric antigen receptor (CAR)-T cells are increasingly used for the treatment of hematologic malignancies. Treatment success relies highly upon sufficient expansion of CAR-T effector cells. Accordingly, longitudinal quantification of CAR-T cells during therapy is clinically important. Techniques to quantify CAR-T cells in patient blood samples are based on flow cytometry and PCR. However, cellular kinetics of CAR-T cells are very complex and under current investigation. In this study, feasibility of CAR-T cell quantification by cell-free DNA (cfDNA) was analyzed. cfDNA isolated from 74 blood samples of 12 patients during lymphoma treatment with the anti-CD19 CAR-T cell product axicabtagene ciloleucel (axi-cel) were analyzed. Concentrations of cfDNA specific for the CAR-T gene construct (cfCAR-DNA) and a reference gene were quantified by a newly designed digital-droplet PCR (ddPCR) assay. Detection and quantification of cfCAR-DNA was feasible and reliable for all patients included. Relative quantification of cfCAR-DNA compared to a reference gene, suitable for genomic DNA analysis, was heterogeneous in treatment responders and non-responders. In contrast, parallel analyses of cfCAR-DNA and reference cfDNA in a patient-specific approach gave insight into active lymphoma killing and treatment responses. In summary, plasma cfDNA determination in lymphoma patients is a promising tool for future clinical decision making.

INTRODUCTION

Chimeric antigen receptor (CAR)-T cells are increasingly used for the treatment of hematologic malignancies. Currently, CAR-T cells are approved in B cell neoplasia including diffuse large B cell lymphoma (DLBCL), mantle-cell lymphoma, B-lineage acute lymphatic leukemia (B-ALL), and multiple myeloma.[1–4] In most studies, sufficient expansion of the CAR-T cells is associated with overall response rates and side effects, such as cytokine-release syndrome (CRS) and immune effector cell-associated neurotoxicity syndrome (ICANS).[5–8] Thus, quantification of CAR-T cells in patients during treatment is important in research and routine clinical settings.

Techniques including flow cytometry and qPCR are commonly used to detect CAR-T cells in patients' blood samples.[1,2,6,9] In these studies, CAR-T cell expansion followed a distinct pattern, reaching a maximum peak within the first 7 to 14 days, followed by subsequent reduction and loss of CAR-T cell signal in a proportion of patients.[1,6] Recently, our group and others have developed digital-droplet PCR (ddPCR) assays to detect CAR-T cells in peripheral blood.[10,11] The advantages of ddPCR compared to quantitative real-time PCR (qPCR) are high interlaboratory reproducibility, omission of calibration curves, and also feasibility of the assay.[12,13] Moreover, ddPCR assays have low limits of detection and are already applied in multiple settings, including analyses of chimerism and minimal residual disease.[12,14] The handling of qPCR and ddPCR data is currently discussed, because multiple factors influence the amount and composition of cellular (genomic) DNA in patients' blood samples during CAR-T therapy.[15,16]

Following re-infusion, CAR-T cells distribute into various body areas and actively migrate into both tumor stroma and lymph nodes.[16] It is unknown whether loss of detectable CAR-T cells in blood samples corresponds to a loss of CAR-T cells due to apoptosis by cellular exhaustion or if it reflects cell sequestration in targeted tissues. Currently, studies investigating the biology of CAR-T cells following therapeutic administration are mainly focused on cellular samples. However, better understanding of CAR-T cell proliferation and distribution in patients could help to optimize long-term success of CAR-T treatments.

Received 4 June 2021; accepted 26 October 2021;
https://doi.org/10.1016/j.omtm.2021.10.009.

Correspondence: Roland Schroers, MD, Ruhr-University Bochum, In der Schornau 23-25, D-44892 Bochum, Germany. E-mail:
E-mail: Roland.Schroers@rub.de
Correspondence: Susanne Klein-Scory, PhD, Ruhr-University Bochum, In der Schornau 23-25, D-44892 Bochum, Germany. E-mail:
E-mail: Susanne.Klein-Scory@rub.de

This is an open access article under the CC BY license (http://creativecommons.org/licenses/by/4.0/).

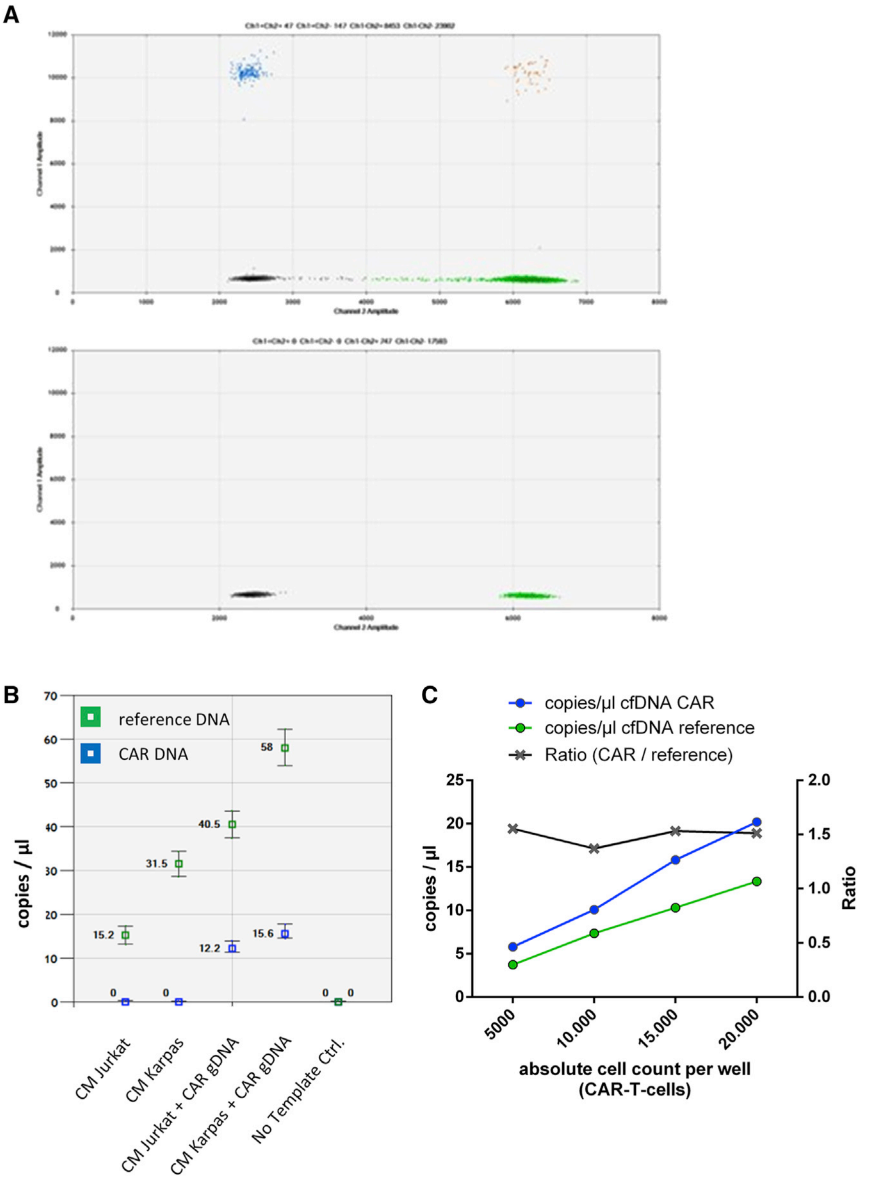

Figure 1. ddPCR assay and *in vitro* experiments

(A) Two-dimensional plots of ddPCR analysis. Two cfDNA samples from patients were analyzed by CARTP-A1 primer-probe pair. Top: Peripheral Blood sample from a patient treated with axi-cel. Bottom: Sample from a patient not treated with axi-cel. black: negative droplets, green: Hex (reference) positive droplets, blue: FAM (CAR-DNA) positive droplets, orange: FAM + Hex double-positive droplets. (B) Spike-in experiments. gDNA of CAR-T cells (2 ng/µL, isolated from the leftovers of an infusion bag) was spiked into DNA collected from CM of Jurkat and Karpas cell lines. No false positive signals were observed. The amount of reference cfDNA was higher in CM obtained from Karpas cells, most likely due to higher rates of apoptosis in the cell culture. In spike-in experiments, reference DNA increased concomitant to CAR-DNA, as the spiked gDNA comprises both the CAR-DNA and reference DNA. Green: Hex-positive droplets (*TERT* reference), blue: FAM-positive droplets (CAR-DNA). (C) Analysis of CM from increasing amounts of cultured CAR-T cells. Cells were seeded in 200 µL medium and cultured for 24 h. Numbers of CAR-T cells correlated with cfCAR-DNA (r^2 = 0.99). Increasing amounts cfCAR-DNA occured concurrently with increasing amounts of reference DNA. Every CAR-T cell harbored the CAR-DNA and the reference DNA (*TERT*), which were both released from apoptotic CAR-T cells. Accordingly, the ratio of CAR-DNA and reference DNA was constant.

Quantification of cell-free DNA (cfDNA) may provide further insight into CAR-T cell kinetics and distribution. Specifically, cfDNA analysis in liquid biopsies represents a modern tool to routinely examine genetic features and treatment responses in various solid tumors.[17,18] cfDNA comprises DNA derived from several areas within the body, including tumor sites and the central nervous system.[19] Accordingly, we hypothesized that CAR-T cells inducing tumor cell death would contribute to the pool of cfDNA in peripheral blood.

Here, we report the detection of CAR-T cell-derived cfDNA *in vitro* and in peripheral blood samples. This is the first study using liquid biopsies to analyze *in vivo* effector cell kinetics in lymphoma patients during CAR-T therapy.

RESULTS

Detection of cell-free CD19-CAR-DNA derived from CAR-T cells

We adjusted our recently published ddPCR assay in order to detect cfDNA. Since cfDNA fragments in liquid biopsies are shorter,[20] primers were chosen to cover smaller amplicons (Figure S1). The modified ddPCR assay was evaluated by analyzing cfDNA obtained from patients treated with axi-cel and also by analyzing conditioned medium (CM) in cell culture experiments. cfDNA was reliably detected with the primer-probe pairs used, which distinctly discriminated between FAM-labeled (CD19-CAR) and Hex-labeled (*TERT* as reference gene) events in patients' cfDNA samples (Figure 1A). The amount of TERT correlated well with the absolute amount of analyzed cfDNA in patients' samples (r^2 = 0.93, Figure S2). Moreover, detection of CD19-CAR fragmented DNA was demonstrated in spike-in experiments without interference by CM collected from Karpas422 and Jurkat cultures (Figure 1B). As expected, higher cell numbers led to higher amounts of reference cfDNA, as it reflected the amount of total cfDNA. Additional experiments including titration, reproducibility, and precision analyses proved the analytical performance of the modified ddPCR assay (Figures S3, S4, and S5). The assay had a limit of detection of at least 3 CAR-T cells in a background of 10,000 cells when used for cellular DNA analysis (0.03%; Figure S5A). Patient-derived cfCAR-DNA from a patient treated with axi-cel was reliably detectable in a genomic DNA (gDNA) background, with a limit

of detection between 0.1% and 0.03% (Figure S5B). We calculated that at least 77 copies of the CAR transgene per mL plasma were reliably detectable with our assay.

cfDNA originates mainly from apoptotic cells.[21] With every apoptotic cell, reference (*TERT*) DNA is released and measurable in the cfDNA pool, but only CAR-T cells release the *axi-cel* DNA. As high transduction efficacies of T cells are achieved during the manufacturing process, some T cells harbor several CAR transgenes, as viral integration follows the Poisson distribution.[22,23] Thus, the mean number of transgenes per T cell could be higher than the number of reference genes, causing a ratio of >1 when compared with these genes in PCR analysis.

To establish a correlation of CAR-T cell numbers and copies/μL of cfCAR-DNA, we analyzed CM collected from cultures with different cell numbers that had been incubated for 24h. We found that increased cell numbers resulted in increased copies/μL of cfCAR-DNA and of reference cfDNA (r^2 = 0.99). Accordingly, the ratio remained constant at ≈1.5 (Figure 1C). In summary, CAR-T cell numbers and thus CAR-T cell expansion, could be detected in parallel analysis of reference cfDNA and specific cfCAR-DNA.

CD19-CAR cfDNA during treatment with axi-cel

Next, cfDNA in peripheral blood samples from patients treated with axi-cel was analyzed over time. Overall, 74 blood samples were collected from 12 lymphoma patients after axi-cel infusion at various time points. Follow-up ranged from 3 to 12 months after infusion of axi-cel. Patients' characteristics and clinical courses are shown in Table 1. We focused on relative quantification of the ratio of cfCAR-DNA to the reference gene first.

cfCAR-DNA was detectable shortly after CAR-T treatment in most patients (Figures 2A and 2B). We interpreted this finding as cfDNA infused with the CAR-T cell product. In patients with tumor responses after axi-cel infusion, it was found that the ratio of cfCAR-DNA increased during the first days after treatment (Figure 2A). Strikingly, all patients responding to axi-cel showed a peak of cfCAR-DNA ratio around day 10 post infusion.

In patients with disease progression within 6 months after axi-cel treatment, the ratio of cfCAR-DNA had a heterogeneous course (Figure 2B). In samples from patient 3, a very low ratio (<0.5%) was measured. In contrast, samples from patients 7 and 12 showed a higher maximum ratio (4% and 8%) compared with some treatment responders (Figure 2A, e.g., patient 5). Patients 9 and 12 were initially responding to the treatment, but eventually relapsed.

Taken together, the ratio of cfCAR-DNA in the analyzed samples was not significantly different between responders and non-responders (p = 0.561 and p = 0.109) (Figure S6). The variation within both groups was high. Also, neither peak of the ratio, nor the area under the curve correlated with the grade of CRS, ICANS, or clinical response (*data not shown*). Thus, analyzing the ratio in cfDNA samples was unsatisfying.

Amount of CD19-CAR cfDNA in relation to reference cfDNA during therapy

To better understand the dynamics of cfDNA, we further considered the individual courses of cfCAR-DNA and cfDNA of the reference gene (*TERT*) in all patients included in the study (Figure 3). As expected, high inter-individual variations for the amounts of cfCAR-DNA and reference cfDNA were noticed. In all samples of those patients responding to axi-cel treatment, increases of cfCAR-DNA concentrations were observed (Figure 3A). In patients 5, 8, and 11, the increments of cfCAR-DNA and the reference cfDNA were simultaneous. In patients 1, 2, 4, 6, and 10, the increase of the reference cfDNA was delayed.

On the contrary, absolute copies/μL of reference cfDNA were immediately declining after transfusion of axi-cel in patients with lymphoma progression during short-term follow-up (Figure 3B). In 2 of 4 non-responders, no increase of cfCAR-DNA was detected (Figure 3B, patients 3 and 9). In patients 7 and 12, cfCAR-DNA rose without robust increase of the reference cfDNA (Figure 3B). Along with disease progression, reference cfDNA rose in patient 3 without an increase of cfCAR-DNA (Figure 3B). This may be explained by a higher lymphoma burden, which can cause increasing amounts of reference cfDNA.[25] The same phenomenon was observed in patient 12. Initially responding towards treatment, this patient suffered from disease progression shortly after the 3-month follow-up.

Increase of reference cfDNA influences the ratio to cfCAR-DNA in patients responding to axi-cel treatment

Subsequently, analyses of the ratio and the absolute amount cfDNA (copies/μL) for each individual patient were compared. In patients responding to treatment, the increase of absolute cfCAR-DNA was accompanied by a substantial increase of reference cfDNA (Figures 2A and 3A, e.g., patient 5). Although substantial increment of cfCAR-DNA (20–25 copies/μL) was observed in these patients, the ratio of cfCAR-DNA to reference DNA was decreased by the increase of reference cfDNA. In patient 10, increase of reference DNA was accompanied by decrease of the relative quantity of cfCAR-DNA over time. In patients not responding to axi-cel, increase of the cfCAR-DNA ratio was related to a sole increase of the cfCAR-DNA without raised levels of the reference cfDNA (Figures 2B and 3B, e.g., patients 7 and 9).

We concluded that active killing of tumor cells by CAR-T cells was reflected by the course of the reference cfDNA. Following apoptosis of lymphoma cells due to CAR-T cell attack, cfDNA was released, which was displayed in the concentration of the reference cfDNA.

Combined analysis of CD19-CAR and total cfDNA during specific *in vitro* killing

Next, *in vitro* experiments were performed to illustrate the potential of cfDNA quantification to monitor both effector and target kinetics

Table 1. Patients' characteristics, including therapies prior to axi-cel and clinical outcome

Patient no.	Type of disease	Initial diagnosis	1st-line therapy	2nd-line therapy	3rd-line therapy	Apheresis and CAR-T cell transfusion	CRS/ICANS	Follow-up
P 1	DLBCL	06/18	6x R-CHOP (PR)	1x R-DHAP<br>1x R-ICE (PD)	Allo-Tx[+] (PR)<br>(04/19)	Apheresis: 01/20<br>Bridging: R-Pola<br>Transfusion: 02/20	CRS: 1<br>ICANS: 0	3 mo: CR<br>6 mo: CR<br>12 mo: CR
P 2	Transformed FL	06/16 FL<br>04/19 DLBCL	6x R-CHOP (PR)<br>+ Radiation	R-GemOx (PD)		Apheresis: 01/20<br>Bridging: R-Pola<br>Transfusion: 02/20	CRS: 0<br>ICANS: 0	3 mo: PR<br>6 mo: PR<br>12 mo: CR
P 3	DLBCL	02/19	6x R-CHOP (PR)<br>+ Radiotherapy	3x R-DHOx (PD)	3x R-ICE (01/20)	Apheresis: 02/20<br>Bridging: -Transfusion: 03/20	CRS: 1<br>ICANS: 0	3 mo: PD
P 4	DLBCL	04/19	6x R-CHOP<br>+2x HD Mtx (PD)	4x MATRIX (PD)		Apheresis: 02/20<br>Bridging: Radiation<br>Transfusion: 04/20	CRS: 2<br>ICANS: 0	3 mo: PR<br>6 mo: CR
P 5	Transformed FL	03/19 FL<br>06/18 DLBCL	2x R (PR)	6x Pixantron (PR)	Radiatio (04/20)	Apheresis: 04/20<br>Bridging: -Transfusion: 05/20	CRS: 2<br>ICANS: 3	3 mo: CR<br>6 mo: na
P 6	DLBCL	11/11	6x R-CHOP<br>+ Radiation (CR)	3x R-GemOx (PD)		Apheresis: 05/20<br>Bridging: R-Pola<br>Transfusion: 06/20	CRS: 2<br>ICANS: 3	3 mo: CR<br>6 mo: CR
P 7	DLBCL	09/19	6x R-CHOP (PR)	2x R-DHAP (PD)	3x R-ICE 03/20 (SD)	Apheresis: 05/20<br>Bridging: R-Pola<br>Transfusion: 06/20	CRS: 2<br>ICANS: 0	3 mo: PD
P 8	DLCBL	11/19	6x R-CHOP (PR)	2x R-DHOx (PD)		Apheresis: 06/20<br>Bridging: R-Pola<br>Transfusion: 07/20	CRS: 2<br>ICANS: 1	3 mo: PR<br>6 mo: CR
P 9	DLBCL	11/19	1x R-CHOP<br>7x R-CHOEP<br>1x HD Mtx (CR)	2x R-DHAP 06/20 (PD)		Apheresis: 07/20<br>Bridging: Radiation<br>Transfusion: 08/20	CRS: 2<br>ICANS: 0	3 mo: CR<br>6 mo: PD
P 10	DLBCL	01/17	6x R-CHOP (CR)	2x R-DHAP<br>+ auto-Tx* (CR)	Allo-Tx[+]<br>09/18 (CR)	Apheresis: 08/20<br>Bridging: -Transfusion: 09/20	CRS: 1<br>ICANS: 2	3 mo: CR<br>6 mo: CR
P 11	DLBCL	01/19	6x R-CHOP (CR)	3x R-ICE<br>+ auto-Tx* (PR)	Radiation (PD)	Apheresis: 08/20<br>Bridging: R-Benda<br>Transfusion: 10/20	CRS: 2<br>ICANS: 3	3 mo: CR<br>6 mo: CR
P 12	DLBCL	02/19	6x R-CHOP (CR)	3x R-DHAP<br>+ auto-Tx* (PR)	Radiation (PR)<br>09/20	Apheresis 10/20<br>Bridging: -Transfusion: 11/20	CRS: 1<br>ICANS: 3	3 mo: CR<br>6 mo: PD

Cytokine-release syndrome (CRS) and immune effector cell-associated neurotoxic syndrome (ICANS) were graded according to ASTCT guidelines[24]. Allo-Tx = allogeneic stem cell transplantation; Auto-Tx = autologous stem cell transplantation; CR = complete response; DLBCL = diffuse large B cell lymphoma; na = not applicable/lost to follow-up; PD = progressive disease; PR = partial response; SD = stable disease; R-CHOP = rituximab, cyclophosphamide, hydroxydaunorubicin hydrochloride (doxorubicin hydrochloride), vincristine (Oncovin) and prednisone; R-DHAP = rituximab, dexamethasone, high-dose cytarabine (Ara C), cisplatin (platinum); R-ICE = rituximab + ifosfamide + carboplatin + etoposide. R-DHOx = rituximab, dexamethasone, high-dose cytarabine (Ara C), oxaliplatin; FL = follicular lymphoma; DHMtx = high-dose methotrexate; R-Pola = rituximab, polatuzumab; R-GemOx = rituximab, gemcitabine, oxaliplatin; R-CHOEP = rituximab, cyclophosphamide, hydroxydaunorubicin hydrochloride (doxorubicin hydrochloride), vincristine (Oncovin), etoposide, and prednisone. *Conditioning regimen: R-BEAM, [+] Conditioning regimen: Flu-Bu-Cy/ATG.

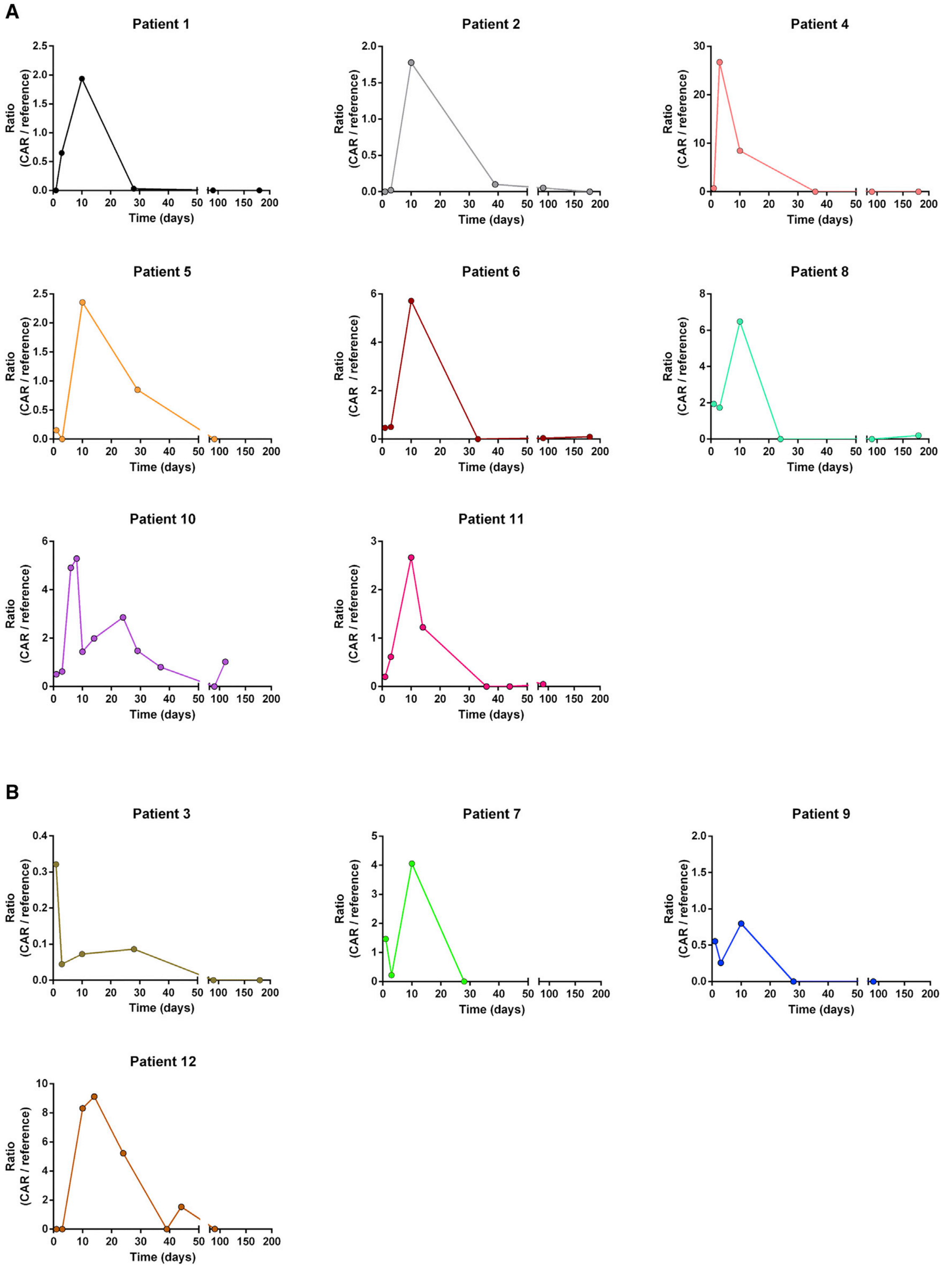

Figure 2. The ratio (%) of cfCAR-DNA to reference cfDNA over time

(A) Follow-up of patients with tumor response after treatment with axi-cel. In these patients, ratio of cfCAR-DNA to reference cfDNA increases within the first days after infusion of CAR-T cells. (B) Follow-up of patients with disease progression after axi-cel treatment. Development of the cfCAR-DNA ratio is heterogeneous. It tends to be lower compared with patients with tumor response in some patients (<1%, patients 3 and 9), but not uniformly (patients 7 and 12).

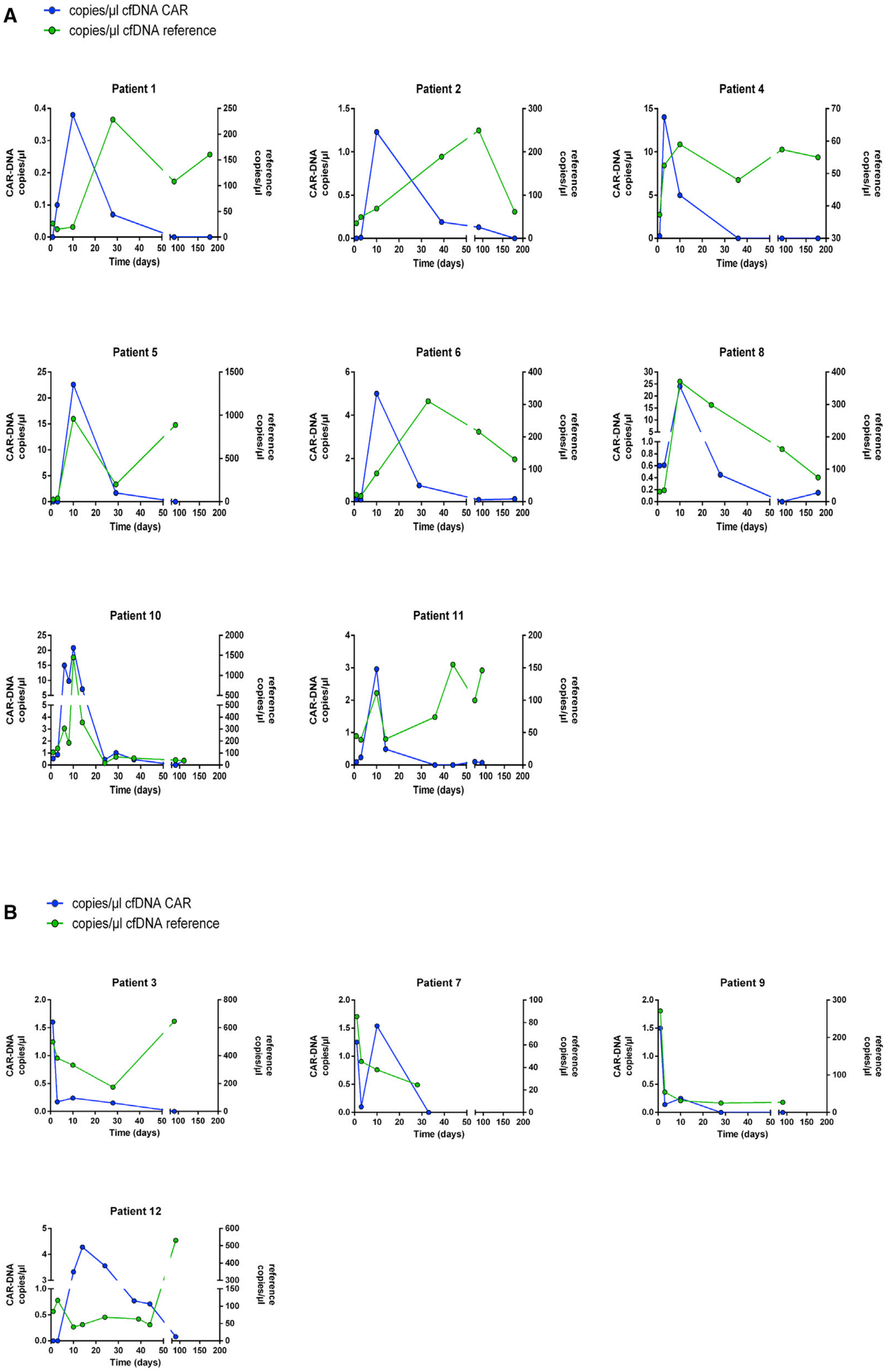

(legend on next page)

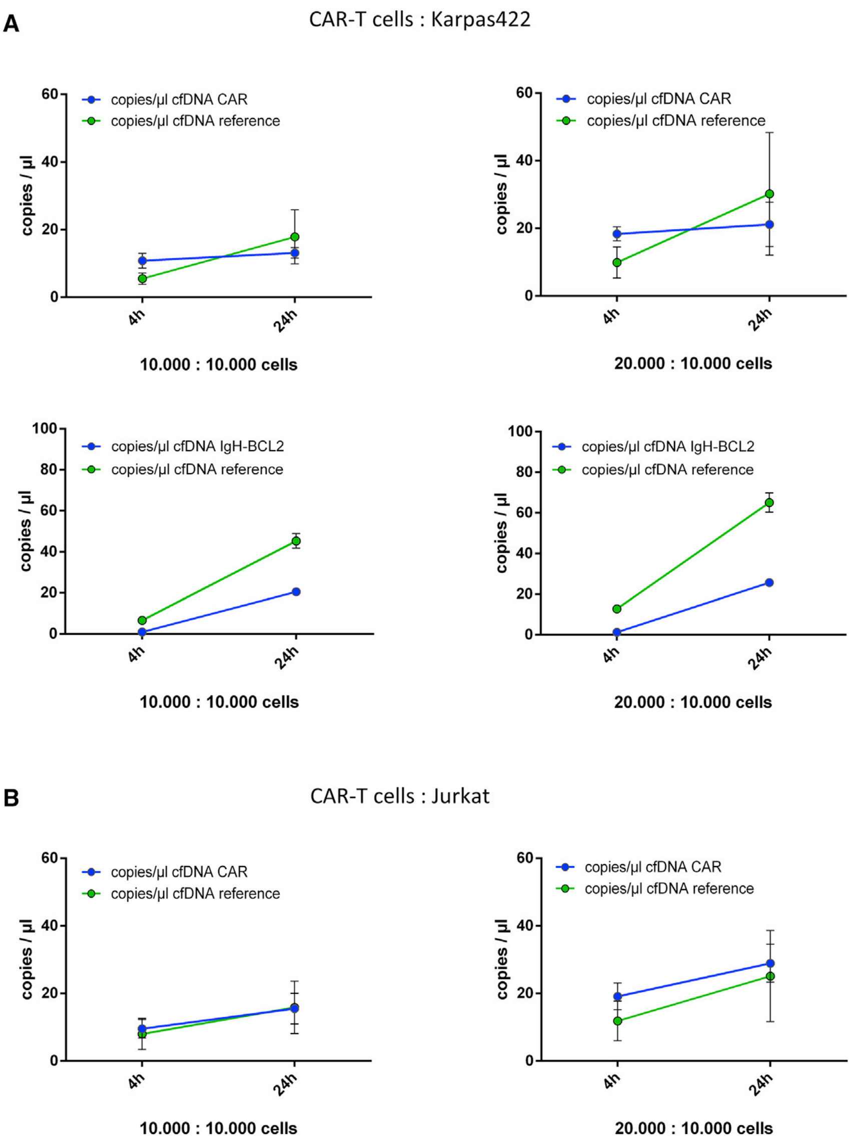

Figure 4. Co-culture experiments of CAR-T cells with Karpas422 and Jurkat cells (n = 4)

Cells were seeded in an effector:target cell ratio of 1:1 (20,000 cells absolute) and 2:1 (30,000 cells absolute) and were cultured for 4 h and 24 h, respectively. (A) CAR-T cells and CD19$^+$ Karpas422. Top: Comparison of cfCAR-DNA and reference cfDNA. Absolute amounts of cfCAR-DNA were higher if more CAR-T cells were seeded (left: 10,000, right: 20,000 CAR-T cells) or due to longer incubation time (CAR-T cell expansion). In both conditions, reference cfDNA (TERT) was lower compared with cfCAR-DNA after 4-h incubation time. After 24-h incubation time, more reference cfDNA was measured, compared with cfCAR-DNA. Bottom: Comparison of IgH-BCL2 cfDNA and reference cfDNA (*PPID*). Concurrently with the reference DNA, Karpas-specific IgH-BCL2 cfDNA increases during incubation of Karpas422 with CAR-T cells. (B) CAR-T cells and CD19$^-$ Jurkat. As expected, the amounts of cfCAR-DNA were again higher if more CAR-T cells were seeded and due to longer incubation time. The amounts of reference cfDNA were not increasing as strong as in (A) and were lower compared with the cfCAR-DNA.

during lymphoma cell killing. CD19$^+$ B cells (Karpas422; 10^4 cells/well) and CD19$^-$ T cells (Jurkat; 10^4 cells/well) were co-cultured with CAR-T cells from patient 10 at effector to target ratios of 1:1 and 2:1, respectively. cfDNA was measured in supernatants following co-culture after 4 and 24h by ddPCR. As shown in Figure 4, the mean concentration of reference cfDNA was 5.5 copies/µL for Karpas and 8.0 copies/µL for Jurkat, respectively (p = 0.33) after 4 h of co-incubation (Figures 4A and 4B, right). Mean cfCAR-DNA concentration was similar (10.8 copies/µL in Karpas and 9.6 copies/µL in Jurkat co-cultures) since both cultures contained approximately 10,000 CAR-T cells. As observed in our previous experiments, absolute copies of cfCAR-DNA were higher when more CAR-T cells were seeded per well (Figures 4A and 4B; 1:1 and 2:1 ratio). In co-cultures of CD19$^+$ Karpas422 cells and CAR-T cells, reference cfDNA increased. Simultaneously with reference cfDNA, Karpas-specific cell-free immunoglobulin (Ig)H-BCL2 (t11; 14) signal increased. The specific killing of Karpas cells resulted in reference cfDNA release and consequently in a decreasing ratio of cfCAR-DNA over time, as described in patient samples (Figure 5, blue columns). However, without specific killing of CD19$^-$ Jurkat cells by CD19-directed CAR-T cells, no additional reference cfDNA was released by Jurkat cells, and, by consequence the ratio of cfCAR-DNA and reference cfDNA remained constant (Figure 5; red columns).

In summary, active killing of lymphoma cells by CAR-T cells was represented by combined analysis of cfDNA derived from effector (CAR-T cells) and target cells (lymphoma cells).

Figure 3. Absolute copies/µL of cfCAR-DNA and reference cfDNA (*TERT*) over time

(A) Follow-up of patients with tumor response after treatment with axi-cel. In all patients, increases of cfCAR-DNA were associated with increasing amounts of reference DNA. (B) Follow-up of patients with disease progression within 6 months after axi-cel treatment. Absolute copies/µL of reference DNA showed an immediate decrease in 3 of 4 patients. An increase of cfCAR-DNA in patients 7 and 12 was not accompanied by a substantial increase of reference DNA. With disease progression, reference cfDNA increased in patients 3 and 12.

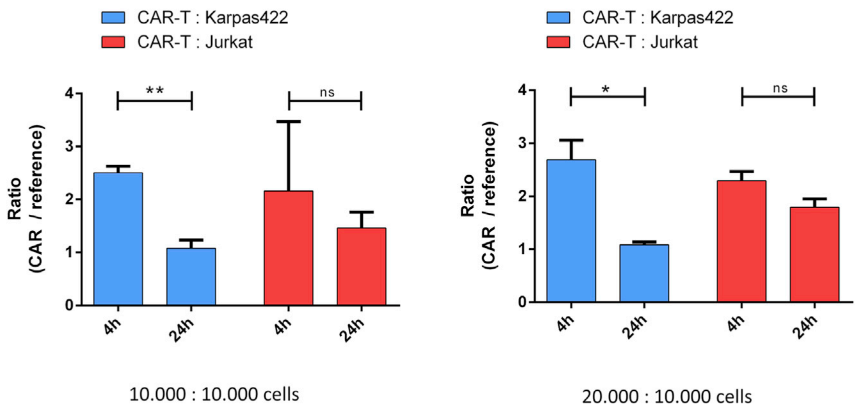

Figure 5. Ratio of cfCAR-DNA in co-culture experiments of CAR-T cells and target cells

The ratio of cfCAR-DNA to reference cfDNA decreased over time due to overshooting increase of reference cfDNA in co-cultures with $CD19^+$ cells (blue columns). In co-cultures with $CD19^-$ Jurkat cells, the ratio was constant (red columns).

Stability of cell-free CD19-CAR-DNA and relation to renal function

In addition to reproducibility and reliability of cfDNA tests, the *in vitro* stability of the cfDNA analytes is an important assay aspect. Accordingly, we investigated whether cfCD19-CAR-DNA concentrations were stable in the collection tubes. Three separate blood samples were collected at the same time from the same patient. cfDNA was isolated after 2h, 48h, and 72h, respectively. The concentrations (copies/μL) of cfCAR-DNA and reference cfDNA did not differ significantly between samples, which indicated cfDNA stability over a minimum of 3 days (Figure S7).

Because cfDNA is eliminated by renal excretion, serum creatinine levels in 3 patients were analyzed in relation to the ratio of cfCAR-DNA to reference cfDNA. The initial increases of cfCAR-DNA were not accompanied by a change in renal function (Figure S8). Also, the amount of cfCAR-DNA did not correlate with serum creatinine levels (r^2 = 0.004). In summary, no alteration of cfCAR-DNA levels, which may potentially influence quantitative results, was observed in our study.

DISCUSSION

For the first time, this study demonstrates the feasibility of CAR-T cell quantification in patients treated with axi-cel by analysis of cfDNA using ddPCR. We proved that CAR-T cells significantly contribute to the pool of circulating cfDNA in patients treated with axi-cel and that CAR-T cell expansion is displayed in the relative amount of cfCAR-DNA. The kinetics and origin of cfDNA are still under investigation, but it is generally accepted that apoptotic cells contribute to the release of cfDNA in a major fashion.[25,26] Assuming that the proportion of apoptotic cells in the CAR-T cell population is constant, higher CAR-T cell numbers, e.g., due to proliferation, lead to increasing amounts of specific cfCAR-DNA. This assumption is supported by the correlation of absolute CAR-T cell counts and amount of cfCAR-DNA in the cell culture experiments performed in this study. Concordantly, higher levels of cfCAR-DNA were measured in patients responding to axi-cel treatment. In summary, cfCAR-DNA could be used as a surrogate for absolute CAR-T cell counts *in vivo*.

Based on our previous observations analyzing cellular CAR-DNA,[10,17] we first focused on the ratio of cfCAR-DNA in cfDNA of patients treated with axi-cel. Notably, increasing amounts of total cfDNA, as demonstrated by increasing reference cfDNA, significantly influenced the ratio of cfCAR-DNA to reference DNA within the first days after axi-cel infusion. We postulated that this increase of reference DNA was likely due to death of lymphoma cells attacked by CAR-T cells. Recently, it has been demonstrated that the release of cfDNA from tumor cells, mainly due to necrosis and apoptosis, increases during cancer treatment.[26] Adoptive cell therapy and immunotherapy were not analyzed in this study in detail; however, it seems likely that tumor cells killed by CAR-T cells release similar amounts of cfDNA. Our hypothesis is supported by the *in vivo* and *in vitro* findings in this study. Inter-individual courses of cfDNA were heterogeneous in responders and non-responders. We found increasing amounts of reference cfDNA (*TERT*) related to the increase of cfCAR-DNA in patients who responded to axi-cel treatment. The ratio of cfCAR-DNA was reduced by a significant increase of reference cfDNA in these samples. On the contrary, in patients with disease progression, this was not the case. In 2 patients (patients 9 and 12), no detectable tumor was obvious in the disease staging 3 months after treatment, but both had disease progression shortly thereafter. In patient 9, cfCAR-DNA was not increasing at all, whereas cfCAR-DNA increased in patient 12 without substantial increase of reference cfDNA. The reason for initial disease remission remains unclear in both patients. Lymphodepleting chemotherapy may be responsible for the initial remission. With ongoing disease progression, total amount of cfDNA increased without an increase of cfCAR-DNA in 2 patients (patients 7 and 12). Interestingly, absolute amounts of cfDNA are higher in patients with lymphoma or other tumors, compared with healthy individuals.[21,25,27]

As shown in cell culture experiments, CAR-T cell-induced killing led to the expected increases of cfDNA. In co-cultures of $CD19^+$ Karpas422 and CAR-T cells, we found a decreased ratio of cfCAR-DNA to reference cfDNA, mainly due to an increase of reference cfDNA (*TERT*) released by dying $CD19^+$ target cells. We confirmed our assumption by the analysis of tumor-specific IgH-BCL2 cfDNA (t 14;18) in CM. If $CD19^-$ Jurkat cells were co-incubated with CAR-T cells, cfCAR-DNA and reference cfDNA increased less and simultaneously. Accordingly, the ratio remained constant. Based on these *in vitro* and *in vivo* observations, we concluded that individual courses of the cfCAR-DNA, the reference

cfDNA, and the ratio indicated the treatment outcomes in patients treated with axi-cel.

In previous studies, in which specific cell-free tumor DNA (circulating tumor DNA [ctDNA]) in response to conventional chemotherapy or radiotherapy was analyzed, decreases of ctDNA were associated with tumor response and indicative of favorable survival.[17,21] A recent study on the origin of cfDNA emphasized the complex interplay among senescence, necrosis, and apoptosis during cancer treatment. As expected, anti-tumor treatments can result in caspase activation and apoptosis, both resulting in release of cfDNA. In contrast, other therapies cause cytostatic effects and cellular senescence without the release of cfDNA.[26] Cytotoxic T cells induce apoptosis rapidly via FAS-Ligand and the perforin/granzyme pathway. These pathways increase caspase activity in the target cells and promote cfDNA release.[28,29] Thus, different treatment modalities obviously can influence the release of cfDNA. To the best of our knowledge, no prior study has investigated the short-term course of cfDNA in the setting of cellular immunotherapy such as CAR-T cell therapy.

In our study, a detailed follow-up within the first days after CAR-T cell infusion revealed significant changes in cfDNA during CAR-T cell treatment during the first days after infusion (Figure 3 A, patient 10). This suggested that a substantial increase of reference cfDNA and specific cfCAR-DNA may display treatment success in CAR-T cell therapy. As the success of CAR-T cell treatment in aggressive lymphoma is determined early after infusion in most patients,[1] understanding the course of cfDNA potentially could improve therapeutic strategies by timely intervention .

cfDNA is of increasing interest to predict responses toward lymphoma treatment.[21,30] Liquid biopsy enables follow-up of specific mutations during cancer treatment.[17,18] The genetic landscape of aggressive lymphoma subtypes is heterogeneous. Accordingly, follow-up of specific mutations is challenging and there is no established method in clinical practice to monitor ctDNA during lymphoma treatment, yet.[30] PCR-based techniques, such as ddPCR or qPCR are under intensive investigation, but face major challenges.[30] Next generation sequencing also facilitates analyses of cfDNA. However, these approaches thus far are not applicable in day-by-day monitoring. One limitation of our study is the missing follow-up of specific lymphoma mutations. Accordingly, the increase of reference cfDNA in the analyzed blood samples may be caused by infections or other pathologic conditions,[31,32] and not by apoptotic tumor cells. Because of the coincidence in time of CAR-T cell expansion and the increase of reference cfDNA, we consider this to be rather unlikely.

CAR-T cell expansion and persistence are major factors in CAR-T cell treatment, but treatment response is dependent on multiple factors, explaining incongruent results between CAR-T cell kinetics and treatment response.[33] To further understand the kinetics of cfDNA in cellular immunotherapy, simultaneous investigation of CAR-T and lymphoma-derived cfDNA during treatment will be of interest in future studies.[34] Particularly, cfDNA analysis is attractive to investigate both lymphoma-derived DNA and cfCAR-DNA, as it contains DNA fragments from all areas of the body.

Currently, clonal kinetics as well as *in vivo* distribution of CAR-T cells after re-infusion are topics of research.[16,35,36] Analyzing blood cells by flow cytometry and quantification of genomic DNA in peripheral blood mononuclear cells are the current standards to quantify CAR-T cells during treatment. Assay-specific limitations in qPCR analysis are adapted and under investigation.[15] Certain limitations, such as calculation of obligatory calibration curves, can be resolved by ddPCR.[12] Infiltration of the tumor potentially influences CAR-T cell amount detectable in peripheral blood, especially in lymphoma and in the situation of CNS involvement. Thus, quantification of CAR-Ts in blood samples display CAR-T cell biology and distribution only in part. In contrast, analysis of cfDNA may reflect CAR-T cells in all areas of the body overcoming the limitations of cellular kinetics in blood samples. Moreover, lymphodepleting chemotherapy heavily reduces the cell counts in patients receiving CAR-T cells. Thus, the amount of extracted cellular DNA from blood samples leads to intra- and inter-individual differences. The impact of lymphodepleting chemotherapy on cfDNA kinetics remains unclear, yet. Further studies are needed to evaluate short-term kinetics of cfDNA in the context of immunotherapy. Other methods to improve CAR-T cell follow-up, such as immuno-positron emission tomography (iPET) have recently been reported.[37] However, this approach has several limitations, e.g., heterogeneous sensitivity and limited tracking of CAR-T cells in organs with high blood volume, including heart, liver, and spleen.

In summary, detection of CAR-T cell-derived cfDNA by ddPCR in patients treated with axi-cel was reliable, and cell-free CAR-DNA was stable for a minimum of 3 days in blood collection tubes. CAR-T cells collected in the blood samples were not releasing further cfDNA after collection, thereby causing a false increase of cfCAR-DNA. Since cfDNA is eliminated via the kidneys, hemodialysis or severe acute kidney failure may influence cfDNA concentrations[38]; neither was observed in our study cohort.

Monitoring of specific cfCAR-DNA in combination with the amount of reference cfDNA is a novel technique to predict responses during CAR-T cell therapy in lymphoma patients. Of note, our study proves that the relative quantification of CAR-T cells compared with a reference gene, as it is suitable for gDNA analysis, can be misguiding. However, a patient-specific approach analyzing cfCAR-DNA and reference cfDNA may provide a promising tool in future clinical decision making. We believe these results to encourage further studies investigating the role of cfDNA in CAR-T cell therapy.

MATERIALS AND METHODS

Patient information

Blood samples were obtained from patients who were treated with axi-cel at two German treatment centers (Knappschaftskrankenhaus Bochum, Department of Hematology and Oncology and Universitätsmedizin Göttingen, Clinic for Hematology and Medical

Oncology). All patients had measurable disease at the time of axi-cel treatment. Treatment was carried out according to the manufacturer's instructions with lymphodepleting chemotherapy comprising fludarabine (30 mg/m^2) and cyclophosphamide (500 mg/m^2). Patients had given informed consent, and the study was approved by the local ethics committee (#19–6750). Patient information is summarized in Table 1.

Cell culture experiments

For cell culture experiments, all CAR-T cells in the infusion bag that remained after axi-cel administration were collected, cultured, and expanded in 96-well round-bottom plates. T cells were washed twice with PBS and cultured in Roswell Park Memorial Institute medium (RPMI1640) + 10% fetal calf serum (FCS) + 1% penicillin/streptomycin +200 IU/mL interleukin-2. To obtain conditioned media, supernatants were carefully collected without disturbing the cell pellet and subsequently centrifuged twice for 5 min at 800 × *g* and then once for 10 min at 2,000 × *g*. Killing assays were performed using the B cell line Karpas422 as target cells. Target cells were cocultured with CAR-T cells in 2 different target:effector cell ratios (1:1; 2:1) in 96-well plates. T-lineage Jurkat cells served as the negative control targets. Experiments were performed in RPMI1640 + 10% FCS + 1% penicillin/streptomycin.

Sample preparation

Blood samples were collected in EDTA tubes and special cfDNA collection tubes (Cell-Free DNA BCT; Streck). Samples were collected from all patients at days 1, 3, 10, and 30 after axi-cel infusion, and where possible at days 90, 180, and 365. Samples were further processed as previously described.[17] Briefly, blood cells were removed by centrifugation at 1,600 × *g* for 10 min. Plasma was carefully collected and stored at −80°C until further use. cfDNA was extracted using a QiaAMP circulating nucleic acid kit (Qiagen, Hilden, Germany), used according to the manufacturer's instructions. The plasma volume was 3 mL and the elution volume was 70 μL. Cellular DNA of the cell pellet was extracted using the QiaAMP blood Mini Kit (Qiagen), used according to the manufacturer's instructions.

PBMCs were isolated from whole blood samples by density gradient centrifugation, as previously described.[10] Cellular DNA extracted from residual cells from an axi-cel infusion bag using the QiaAMP Mini Kit (Qiagen) advanced by RNAse digestion (Qiagen) was used as a positive control. Cells were stored at −80°C until use. DNA isolated from untransduced PBMCs was used as a negative control.

ddPCR for detection of axi-cel and IgH-BCL2 translocation

Axi-cel-specific DNA was amplified from DNA by conventional PCR with primers binding to the long terminal repeats of the retrovirus, as previously described.[10] Primer-probe assays for analysis of cfDNA were designed with Primer 3 software and ordered from BioRad. The resulting amplicon of the primer-probe pair was 77 base pairs in length, which was suitable for assessment in cfDNA assays. Primer-probe sequences were as follows:

Primer-Probe-Assay (CARTP-A1)

F: TGGAAATAACAGGCTCCACC

R: CAGTTTCACCTCGCCCTT

P: CCGGCAAGCCCGGATCTGGCG

Both primers bind to the FMC63-derived sequence within the original construct. Analysis of cellular DNA was carried out as previously described.[10]

Digital-PCR was performed as previously described by our group.[10,12] The reactions were set up in 20-μL sample volumes containing 10 μL of 2 × ddPCR Supermix (no dUTP; BioRad), 1 μL FAM-labeled primer-probe-assay, 1 μL Hex-labeled reference primer-probe assay, 5 μL cfDNA from the 70 μL eluate, and nuclease-free water to adjust the sample to the final volume. For cellular DNA analysis, gDNA was diluted to 10 ng/μL if possible, or used purely if the concentration was below 10 ng/μL. Subsequently 5 μL genomic DNA was used for the PCR reaction and samples were digested with 8U EcoRI restriction enzyme (Fast digest, NEB). HEX-labeled assay (BioRad) detecting reference gene *TERT* was used as a control housekeeping gene for both assays (cfDNA and cellular DNA).[10] The final reaction mixture was incubated for 10 min at 36°C for restriction digestion. Next, droplets were generated in a QX200 droplet generator (BioRad) according to the manufacturer's instructions, giving a final sample volume of 40 μL. For detection of Karpas422-specific IgH-BCL2 translocation (t14; 18), we used an assay previously published,[39] with *PPID* as reference gene. Sensitivity to detect IgH-BCL2 translocation was proofed by low levels of detection (10^{-3}) (Figure S9).

DNA amplification was carried out using the following PCR program: initial denaturation at 95°C for 10 min, 40 cycles of amplification at 94°C for 30 s followed by 60°C for 1 min, and a final denaturation step at 98°C for 10 min (C1000 Touch Thermal Cycler; BioRad). The ramp rate was set to 2.0°C/s. Finally, droplets were analyzed in a QX200 droplet reader (BioRad), and data were processed with QuantaSoft software (BioRad), including Poisson distribution analysis.

Absolute copies/μL and the ratio were calculated by QuantaSoft software.

Handling of ddPCR data

Results of the performed ddPCR assays were expressed as copies/μL. This referred to the copies per μL eluate, by which calculation of copies/μL plasma can be performed. This required a standardized protocol for every sample analyzed, as described above. Copies per milliliter plasma could thus be calculated by the following formula: $copies/\mu l * \frac{20}{5} * \frac{70}{3} = copies/ml\ plasma$.[17,18] We used *TERT* as reference for the total amount of cfDNA in the sample, as every cell (including CAR-T cells) contributing to the pool of cfDNA, in general, releases 2 alleles of *TERT*. The amount of CAR-DNA compared

with the amount of reference DNA in the sample was expressed as the % ratio = $\left(\frac{copies/\mu l\ CAR}{copies/\mu l\ reference}\right) * 100$.

Assay characteristics and statistics

Reproducibility and precision were assessed by replicate tests of cellular DNA obtained from the washout of an axi-cel infusion bag (mean viral copies per genome ≈ 1, assessed by ddPCR) and untransduced T cells (negative control). Limit of blank was calculated based on the replicate tests of a non-template control (aqua).[40] Limit of detection was calculated based on dilution experiments of either CAR-gDNA and patient-derived cfDNA, spiked into reference gDNA.

Regression analysis and Pearson correlation were used for correlation analysis (r^2). All statistical analyses and data plots were carried out with GraphPad Prism software (version 6).

PATIENT INFORMED CONSENT

All patients agreed to participate in this study and gave their written informed consent for blood sample collection, analysis of clinical data, and publication of potentially identifiable information.

SUPPLEMENTAL INFORMATION

Supplemental information can be found online at https://doi.org/10.1016/j.omtm.2021.10.009.

ACKNOWLEDGMENTS

We thank Andrea Reinert and Christina Eilert-Micus for excellent technical support. The study was supported by a FoRUM grant (Ruhr-University Bochum, no. F978-2020).

AUTHOR CONTRIBUTIONS

T.M., S.K.S., A.B., D.V., V.N., and J.T. collected patient samples and performed the experiments. T.M., G.W., S.K.S., and R.S. designed the study and analyzed the data. T.M., S.K.S., and R.S. wrote the manuscript.

DECLARATION OF INTERESTS

The authors declare no conflict of interest.

REFERENCES

1. Neelapu, S.S., Locke, F.L., Bartlett, N.L., Lekakis, L.J., Miklos, D.B., Jacobson, C.A., Braunschweig, I., Oluwole, O.O., Siddiqi, T., Lin, Y., et al. (2017). Axicabtagene ciloleucel CAR T-cell therapy in refractory large B-cell lymphoma. N. Engl. J. Med. *377*, 2531–2544.
2. Maude, S.L., Frey, N., Shaw, P.A., Aplenc, R., Barrett, D.M., Bunin, N.J., Chew, A., Gonzalez, V.E., Zheng, Z., Lacey, S.F., et al. (2014). Chimeric antigen receptor T cells for sustained remissions in leukemia. N. Engl. J. Med. *371*, 1507–1517.
3. Cohen, A.D., Garfall, A.L., Stadtmauer, E.A., Melenhorst, J.J., Lacey, S.F., Lancaster, E., Vogl, D.T., Weiss, B.M., Dengel, K., Nelson, A., et al. (2019). B cell maturation antigen-specific CAR T cells are clinically active in multiple myeloma. J. Clin. Invest. *129*, 2210–2221.
4. Raje, N., Berdeja, J., Lin, Y., Siegel, D., Jagannath, S., Madduri, D., Liedtke, M., Rosenblatt, J., Maus, M.V., Turka, A., et al. (2019). Anti-BCMA CAR T-cell therapy bb2121 in relapsed or refractory multiple myeloma. N. Engl. J. Med. *380*, 1726–1737.
5. Park, J.H., Rivière, I., Gonen, M., Wang, X., Sénéchal, B., Curran, K.J., Sauter, C., Wang, Y., Santomasso, B., Mead, E., et al. (2018). Long-term follow-up of CD19 CAR therapy in acute lymphoblastic leukemia. N. Engl. J. Med. *378*, 449–459.
6. Schuster, S.J., Bishop, M.R., Tam, C.S., Waller, E.K., Borchmann, P., McGuirk, J.P., Jäger, U., Jaglowski, S., Andreadis, C., Westin, J.R., et al. (2019). Tisagenlecleucel in adult relapsed or refractory diffuse large B-cell lymphoma. N. Engl. J. Med. *380*, 45–56.
7. Hirayama, A.V., Gauthier, J., Hay, K.A., Voutsinas, J.M., Wu, Q., Gooley, T., Li, D., Cherian, S., Chen, X., Pender, B.S., et al. (2019). The response to lymphodepletion impacts PFS in patients with aggressive non-Hodgkin lymphoma treated with CD19 CAR T cells. Blood *133*, 1876–1887.
8. Fraietta, J.A., Lacey, S.F., Orlando, E.J., Pruteanu-Malinici, I., Gohil, M., Lundh, S., Boesteanu, A.C., Wang, Y., O'Connor, R.S., Hwang, W.-T., et al. (2018). Determinants of response and resistance to CD19 chimeric antigen receptor (CAR) T cell therapy of chronic lymphocytic leukemia. Nat. Med. *24*, 563–571.
9. Kochenderfer, J.N., Dudley, M.E., Feldman, S.A., Wilson, W.H., Spaner, D.E., Maric, I., Stetler-Stevenson, M., Phan, G.Q., Hughes, M.S., Sherry, R.M., et al. (2012). B-cell depletion and remissions of malignancy along with cytokine-associated toxicity in a clinical trial of anti-CD19 chimeric-antigen-receptor-transduced T cells. Blood *119*, 2709–2720.
10. Mika, T., Maghnouj, A., Klein-Scory, S., Ladigan-Badura, S., Baraniskin, A., Thomson, J., Hasenkamp, J., Hahn, S.A., Wulf, G., and Schroers, R. (2020). Digital-droplet PCR for quantification of CD19-directed CAR T-cells. Front. Mol. Biosci. *7*, 84.
11. Badbaran, A., Berger, C., Riecken, K., Kruchen, A., Geffken, M., Müller, I., Kröger, N., Ayuk, F.A., and Fehse, B. (2020). Accurate in-vivo quantification of CD19 CAR-T cells after treatment with axicabtagene ciloleucel (Axi-Cel) and tisagenlecleucel (Tisa-Cel) using digital PCR. Cancers *12*,, 1970.
12. Mika, T., Baraniskin, A., Ladigan, S., Wulf, G., Dierks, S., Haase, D., Schork, K., Turewicz, M., Eisenacher, M., Schmiegel, W., et al. (2019). Digital droplet PCR-based chimerism analysis for monitoring of hematopoietic engraftment after allogeneic stem cell transplantation. Int. J. Lab. Hematol. *41*, 615–621.
13. Franke, G.-N., Maier, J., Wildenberger, K., Cross, M., Giles, F.J., Müller, M.C., Hochhaus, A., Niederwieser, D., and Lange, T. (2020). Comparison of real-time quantitative PCR and digital droplet PCR for BCR-ABL1 monitoring in patients with chronic myeloid leukemia. J. Mol. Diagn. *22*, 81–89.
14. Link-Lenczowska, D., Pallisgaard, N., Cordua, S., Zawada, M., Czekalska, S., Krochmalczyk, D., Kanduła, Z., and Sacha, T. (2018). A comparison of qPCR and ddPCR used for quantification of the JAK2 V617F allele burden in Ph negative MPNs. Ann. Hematol. *97*, 2299–2308.
15. Yamamoto, S., Matsumoto, S., Goto, A., Ugajin, M., Nakayama, M., Moriya, Y., and Hirabayashi, H. (2020). Quantitative PCR methodology with a volume-based unit for the sophisticated cellular kinetic evaluation of chimeric antigen receptor T cells. Sci. Rep. *10*, 17884.
16. Sugimoto, H., Chen, S., Minembe, J.-P., Chouitar, J., He, X., Wang, H., Fang, X., and Qian, M.G. (2021). Insights on droplet digital PCR-based cellular kinetics and biodistribution assay support for CAR-T cell therapy. AAPS J. *23*, 36.
17. Klein-Scory, S., Wahner, I., Maslova, M., Al-Sewaidi, Y., Pohl, M., Mika, T., Ladigan, S., Schroers, R., and Baraniskin, A. (2020). Evolution of RAS mutational status in liquid biopsies during first-line chemotherapy for metastatic colorectal cancer. Front. Oncol. *10*, 1115.
18. Klein-Scory, S., Maslova, M., Pohl, M., Eilert-Micus, C., Schroers, R., Schmiegel, W., and Baraniskin, A. (2018). Significance of liquid biopsy for monitoring and therapy decision of colorectal cancer. Transl. Oncol. *11*, 213–220.
19. Hattori, K., Sakata-Yanagimoto, M., Suehara, Y., Yokoyama, Y., Kato, T., Kurita, N., Nishikii, H., Obara, N., Takano, S., Ishikawa, E., et al. (2018). Clinical significance of disease-specific MYD88 mutations in circulating DNA in primary central nervous system lymphoma. Cancer Sci. *109*, 225–230.
20. Mouliere, F., Chandrananda, D., Piskorz, A.M., Moore, E.K., Morris, J., Ahlborn, L.B., Mair, R., Goranova, T., Marass, F., Heider, K., et al. (2018). Enhanced detection of circulating tumor DNA by fragment size analysis. Sci. Transl. Med. *10*, eaat4921.
21. Rossi, D., Spina, V., Bruscaggin, A., and Gaidano, G. (2019). Liquid biopsy in lymphoma. Haematologica *104*, 648–652.

22. Fehse, B., Kustikova, O.S., Bubenheim, M., and Baum, C. (2004). Pois(s)on–it's a question of dose. Gene Ther. *11*, 879–881.

23. Mika, T., Ladigan-Badura, S., Maghnouj, A., Mustafa, B., Klein-Scory, S., Baraniskin, A., Döhring, S., Fuchs, I., Ehl, S., Hahn, S.A., et al. (2020). Altered T-lymphocyte biology following high-dose melphalan and autologous stem cell transplantation with implications for adoptive T-cell therapy. Front. Oncol. *10*, 568056.

24. Lee, D.W., Santomasso, B.D., Locke, F.L., Ghobadi, A., Turtle, C.J., Brudno, J.N., Maus, M.V., Park, J.H., Mead, E., Pavletic, S., et al. (2019). ASTCT consensus grading for cytokine release syndrome and neurologic toxicity associated with immune effector cells. Biol. Blood Marrow Transplant. *25*, 625–638.

25. Roschewski, M., Dunleavy, K., Pittaluga, S., Moorhead, M., Pepin, F., Kong, K., Shovlin, M., Jaffe, E.S., Staudt, L.M., Lai, C., et al. (2015). Circulating tumour DNA and CT monitoring in patients with untreated diffuse large B-cell lymphoma: a correlative biomarker study. Lancet Oncol. *16*, 541–549.

26. Rostami, A., Lambie, M., Yu, C.W., Stambolic, V., Waldron, J.N., and Bratman, S.V. (2020). Senescence, necrosis, and apoptosis govern circulating cell-free DNA release kinetics. Cell Rep. *31*, 107830.

27. Gormally, E., Caboux, E., Vineis, P., and Hainaut, P. (2007). Circulating free DNA in plasma or serum as biomarker of carcinogenesis: practical aspects and biological significance. Mutat. Res. *635*, 105–117.

28. Benmebarek, M.-R., Karches, C.H., Cadilha, B.L., Lesch, S., Endres, S., and Kobold, S. (2019). Killing mechanisms of chimeric antigen receptor (CAR) T cells. Int. J. Mol. Sci. *20*, 1283.

29. Karlsson, H., Karlsson, S.C.H., Lindqvist, A.C., Fransson, M., Paul-Wetterberg, G., Nilsson, B., Essand, M., Nilsson, K., Frisk, P., Jernberg-Wiklund, H., et al. (2013). Combining CAR T cells and the Bcl-2 family apoptosis inhibitor ABT-737 for treating B-cell malignancy. Cancer Gene Ther. *20*, 386–393.

30. Rossi, D., Kurtz, D.M., Roschewski, M., Cavalli, F., Zucca, E., and Wilson, W.H. (2020). The development of liquid biopsy for research and clinical practice in lymphomas: report of the 15-ICML workshop on ctDNA. Hematol. Oncol. *38*, 34–37.

31. Saukkonen, K., Lakkisto, P., Pettilä, V., Varpula, M., Karlsson, S., Ruokonen, E., and Pulkki, K. (2008). Cell-free plasma DNA as a predictor of outcome in severe sepsis and septic shock. Clin. Chem. *54*, 1000–1007.

32. Chang, C.P.-Y., Chia, R.-H., Wu, T.-L., Tsao, K.-C., Sun, C.-F., and Wu, J.T. (2003). Elevated cell-free serum DNA detected in patients with myocardial infarction. Clin. Chim. Acta *327*, 95–101.

33. Singh, A.P., Chen, W., Zheng, X., Mody, H., Carpenter, T.J., Zong, A., and Heald, D.L. (2021). Bench-to-bedside translation of chimeric antigen receptor (CAR) T cells using a multiscale systems pharmacokinetic-pharmacodynamic model: a case study with anti-BCMA CAR-T. CPT Pharmacometrics Syst. Pharmacol *10*, 362–376.

34. Pott, C., Brüggemann, M., Ritgen, M., van der Velden, V.H.J., van Dongen, J.J.M., and Kneba, M. (2019). MRD detection in B-cell non-Hodgkin lymphomas using Ig gene rearrangements and chromosomal translocations as targets for real-time quantitative PCR. Methods Mol. Biol. (Clifton, N.J.) *1956*, 199–228.

35. Fraietta, J.A., Nobles, C.L., Sammons, M.A., Lundh, S., Carty, S.A., Reich, T.J., Cogdill, A.P., Morrissette, J.J.D., DeNizio, J.E., Reddy, S., et al. (2018). Disruption of TET2 promotes the therapeutic efficacy of CD19-targeted T cells. Nature *558*, 307–312.

36. Sheih, A., Voillet, V., Hanafi, L.-A., DeBerg, H.A., Yajima, M., Hawkins, R., Gersuk, V., Riddell, S.R., Maloney, D.G., Wohlfahrt, M.E., et al. (2020). Clonal kinetics and single-cell transcriptional profiling of CAR-T cells in patients undergoing CD19 CAR-T immunotherapy. Nat. Commun. *11*, 219.

37. Simonetta, F., Alam, I.S., Lohmeyer, J.K., Sahaf, B., Good, Z., Chen, W., Xiao, Z., Hirai, T., Scheller, L., Engels, P., et al. (2020). Molecular imaging of chimeric antigen receptor T cells by ICOS-ImmunoPET. Clin. Cancer Res.

38. Khier, S., and Lohan, L. (2018). Kinetics of circulating cell-free DNA for biomedical applications: critical appraisal of the literature. Future Sci. OA *4*, FSO295.

39. Drandi, D., Kubiczkova-Besse, L., Ferrero, S., Dani, N., Passera, R., Mantoan, B., Gambella, M., Monitillo, L., Saraci, E., Ghione, P., et al. (2015). Minimal residual disease detection by droplet digital PCR in multiple myeloma, mantle cell lymphoma, and follicular lymphoma: a comparison with real-time PCR. J. Mol. Diagn. *17*, 652–660.

40. Armbruster, D.A., and Pry, T. (2008). Limit of blank, limit of detection and limit of quantitation. Clin. Biochem. Rev. *29*, S49–S52.

OMTM, Volume *23*

Supplemental information

Quantification of cell-free DNA for the analysis of CD19-CAR-T cells during lymphoma treatment

Thomas Mika, Julia Thomson, Verena Nilius-Eliliwi, Deepak Vangala, Alexander Baraniskin, Gerald Wulf, Susanne Klein-Scory, and Roland Schroers

Supplemental Material

Supp. Fig. 1

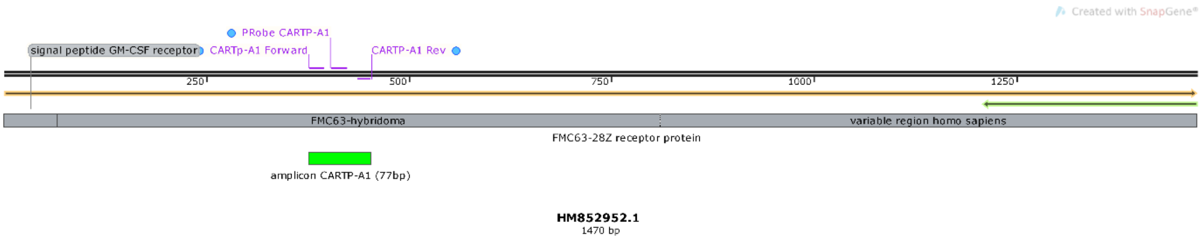

***Supplement Figure 1*: Primer-probe-pair and amplicon within the CD19-CAR gene**

The primer-probe pair to detect cfCAR-DNA was designed to detect an amplicon of 77 bp in length. Reference primer-probe pair was used as previously published [10].

Supp. Fig. 2

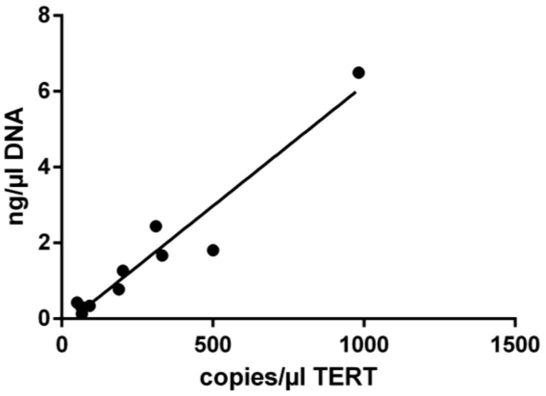

Supplement Figure 2:* Correlation of cfDNA and copies/µl *TERT

Correlation of patients' cfDNA (n=10), isolated from plasma samples, and *TERT*. The amount of cfDNA in the analyzed eluate correlated well with the measured quantity of *TERT* (copies/µl eluate) assessed by ddPCR ($r^2 = 0.93$, $p<0.001$).

Supp. Fig. 3

A

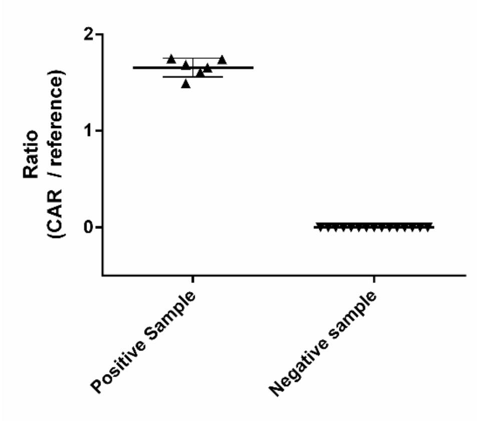

B

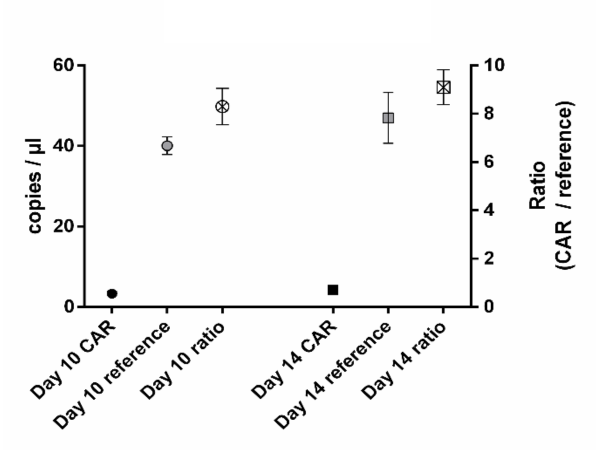

***Supplement Figure 3*: Analytical Performance of the modified ddPCR-assay**

A: Repeated measurements of positive (n = 6) and negative control samples (n = 15) confirmed high reproducibility and precision of the modified ddPCR assay (SD 0.097 and 0.001). Limit of blank was 0.07 copies/µl. For subsequent analyses results below 0.07 copies/µl were set to 0.

B: The reproducibility of the complete workflow of the new ddPCR assay, encompassing DNA isolation from plasma and subsequent PCR amplification in repeated measurements of two blood samples collected from patients treated with axi-cel, was tested. In brief, DNA was isolated from the two samples 4- and 3-times, respectively. High reproducibility of the assay with a standard deviation of the ratio (CAR / reference) of 0.75 and 0.72 was seen in the two experiments.

Supp. Fig. 4

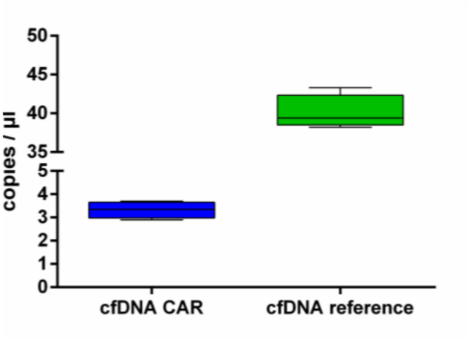

Supplement Figure 4: **Repetitive DNA-isolation from a blood sample 10 days after axi-cel infusion.**

A blood sample from one patient after axi-cel infusion was processed 4-times to obtain cfDNA. Mean copies/µl cfDNA CAR was 3.325 (±0.35). Mean copies/µl reference cfDNA was 40.08 (±2.223).

Supp. Fig. 5

A

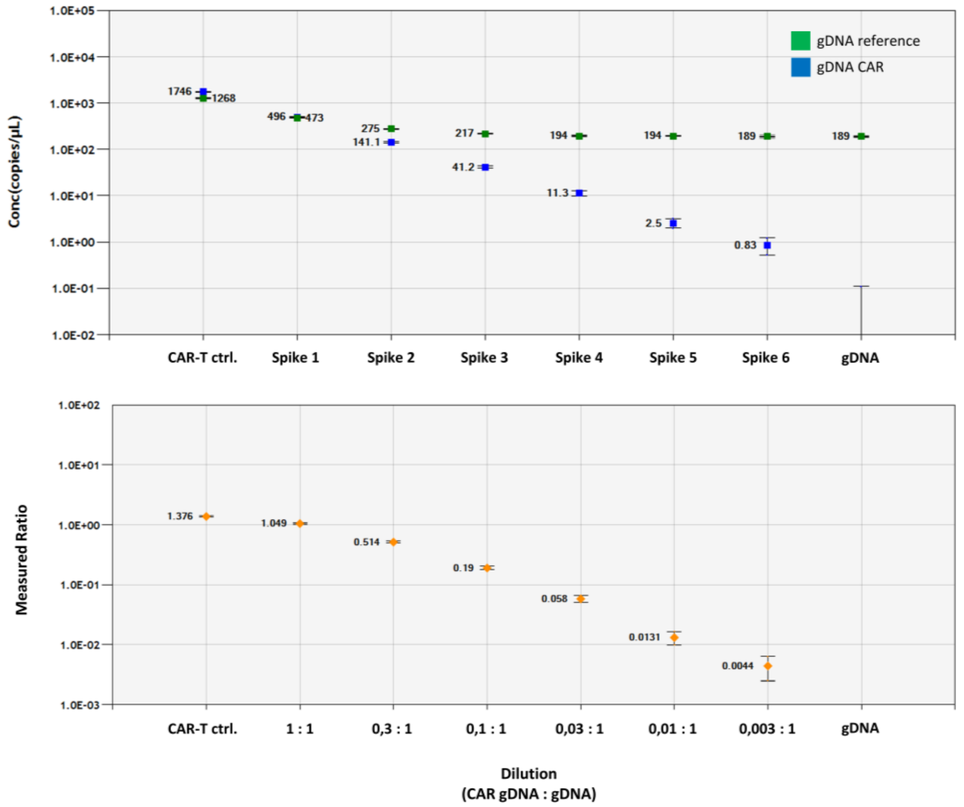
gDNA reference
gDNA CAR
Conc(copies/µL)
1.0E+05
1.0E+04
1.0E+03
1.0E+02
1.0E+01
1.0E+00
1.0E-01
1.0E-02
1746
1268
496
473
275
141.1
217
41.2
194
11.3
194
2.5
189
0.83
189
CAR-T ctrl.
Spike 1
Spike 2
Spike 3
Spike 4
Spike 5
Spike 6
gDNA
Measured Ratio
1.0E+02
1.0E+01
1.0E+00
1.0E-01
1.0E-02
1.0E-03
1.376
1.049
0.514
0.19
0.058
0.0131
0.0044
CAR-T ctrl.
1 : 1
0,3 : 1
0,1 : 1
0,03 : 1
0,01 : 1
0,003 : 1
gDNA
Dilution
(CAR gDNA : gDNA)

B

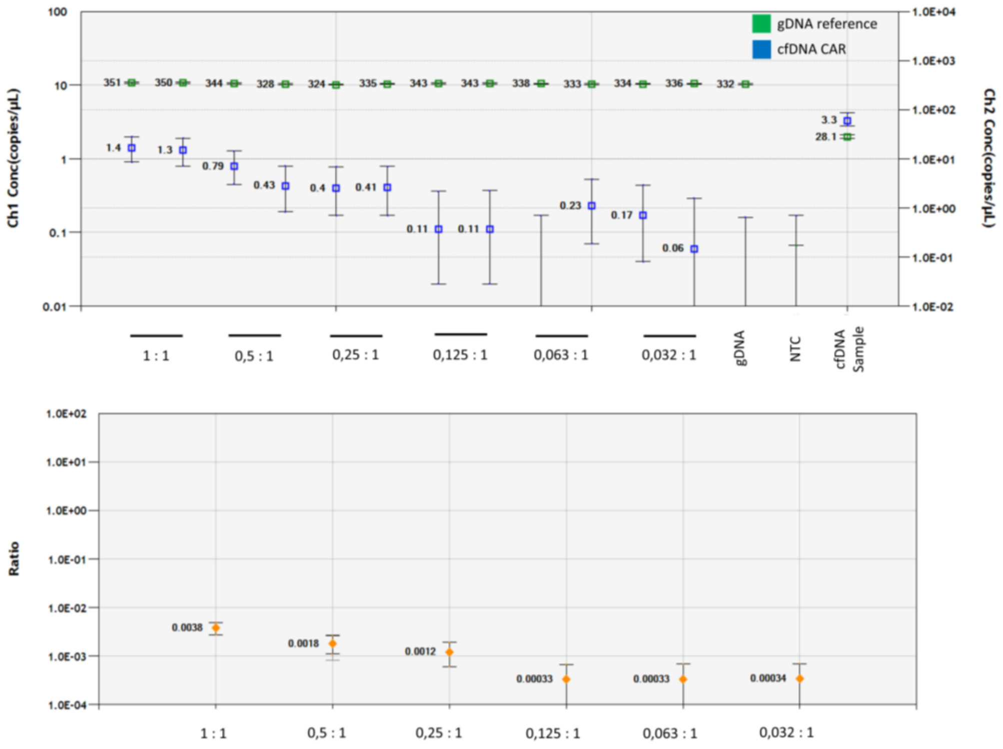
gDNA reference
cfDNA CAR
Ch1 Conc(copies/µL)
Ch2 Conc(copies/µL)
100
10
1
0.1
0.01
1.0E+04
1.0E+03
1.0E+02
1.0E+01
1.0E+00
1.0E-01
1.0E-02
351
350
344
328
324
335
343
343
338
333
334
336
332
1.4
1.3
0.79
0.43
0.4
0.41
0.11
0.11
0.23
0.17
0.06
3.3
28.1
1 : 1
0,5 : 1
0,25 : 1
0,125 : 1
0,063 : 1
0,032 : 1
gDNA
NTC
cfDNA Sample
Ratio
1.0E+02
1.0E+01
1.0E+00
1.0E-01
1.0E-02
1.0E-03
1.0E-04
0.0038
0.0018
0.0012
0.00033
0.00033
0.00034
1 : 1
0,5 : 1
0,25 : 1
0,125 : 1
0,063 : 1
0,032 : 1

Supplement Figure 5: **Limit of Detection of the modified ddPCR-assay**

A: Dilution of CAR gDNA in reference gDNA. For every sample, CAR gDNA (spike1-6) was diluted in a constant amount of reference gDNA (30 ng). At least 0,01 ng CAR gDNA was reliably detectable in a background of 30 ng reference gDNA (0.03%).

B: Dilution of patient derived cfDNA in reference gDNA. For each sample, cfDNA from a patient derived plasma sample was diluted in 40 ng of reference gDNA. cfDNA was reliably detectable in a ratio of 0.1% and still detectable at lower concentrations. Based on the assumption that 308 copies were evident in the cfDNA sample (3.3 cop/µl * (20/5)*(70/3) = 308 copies / ml plasma), limit of detection was between 38.5-77 copies/ml Plasma (0.125 and 0.25 dilution factors).

Supp. Fig. 6

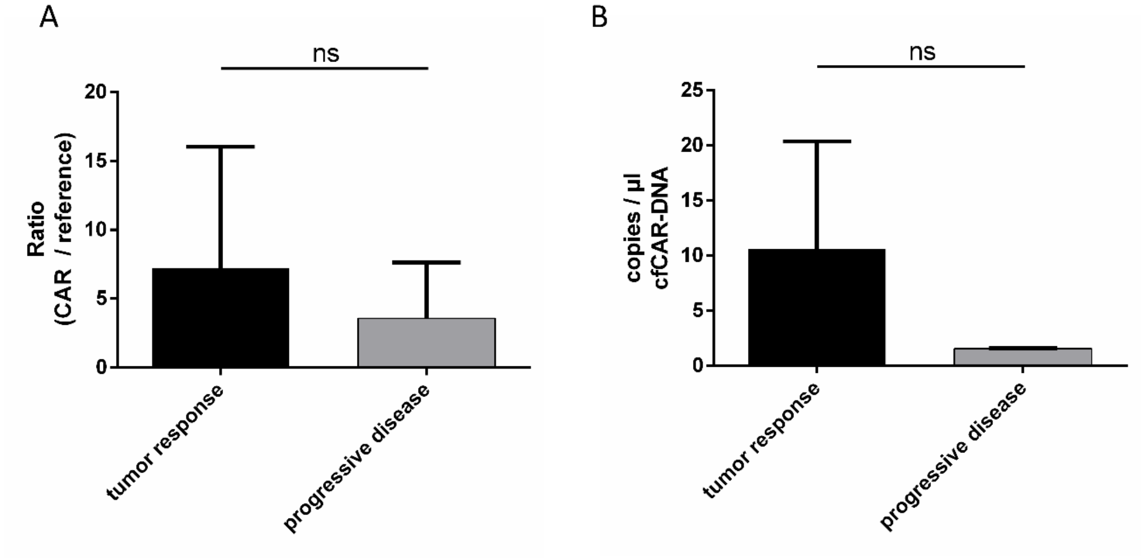

***Supplement Figure 6*: The ratio's peak and the cfCAR-DNAs' peak of patients with tumor response and disease progression.**

A: The ratio's peak of cfCAR-DNA to reference cfDNA tended to be higher in patients responding upon axi-cel treatment. The difference is not significant (p = 0.334). Tumor response: n = 8, progressive disease: n = 4.

B: Maximum absolute amount (copies/µl) of cfCAR-DNA is lower in patients not responding. Difference is not significant (p = 0.151). Tumor response: n = 8, progressive disease: n = 4.

Supp. Fig. 7

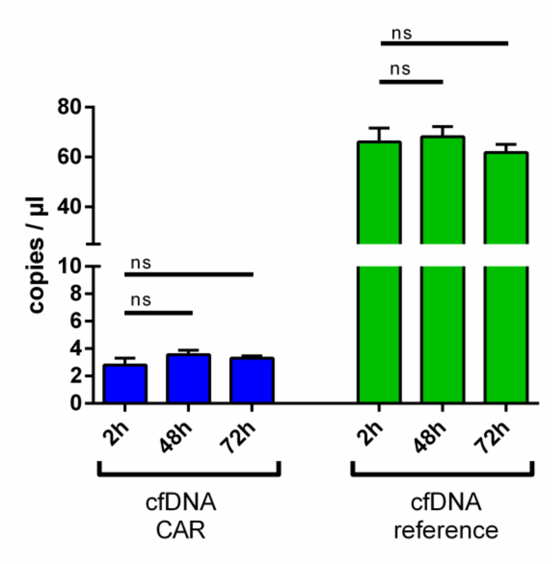

Supplement Figure 7: **Analysis of cfDNA in a patient 14 days after axi-cel infusion.** Three blood samples from one patient at one time point were obtained and further processed after 2 h, 48 h, and 72 h. Each sample was analyzed three times. Absolute copies/µl of cfCAR-DNA and reference cfDNA were constant over time. (cfCAR-DNA; p = 0.089 and p = 0.177, cfDNA reference; p = 0.622 and p = 0.311).

Supp. Fig. 8

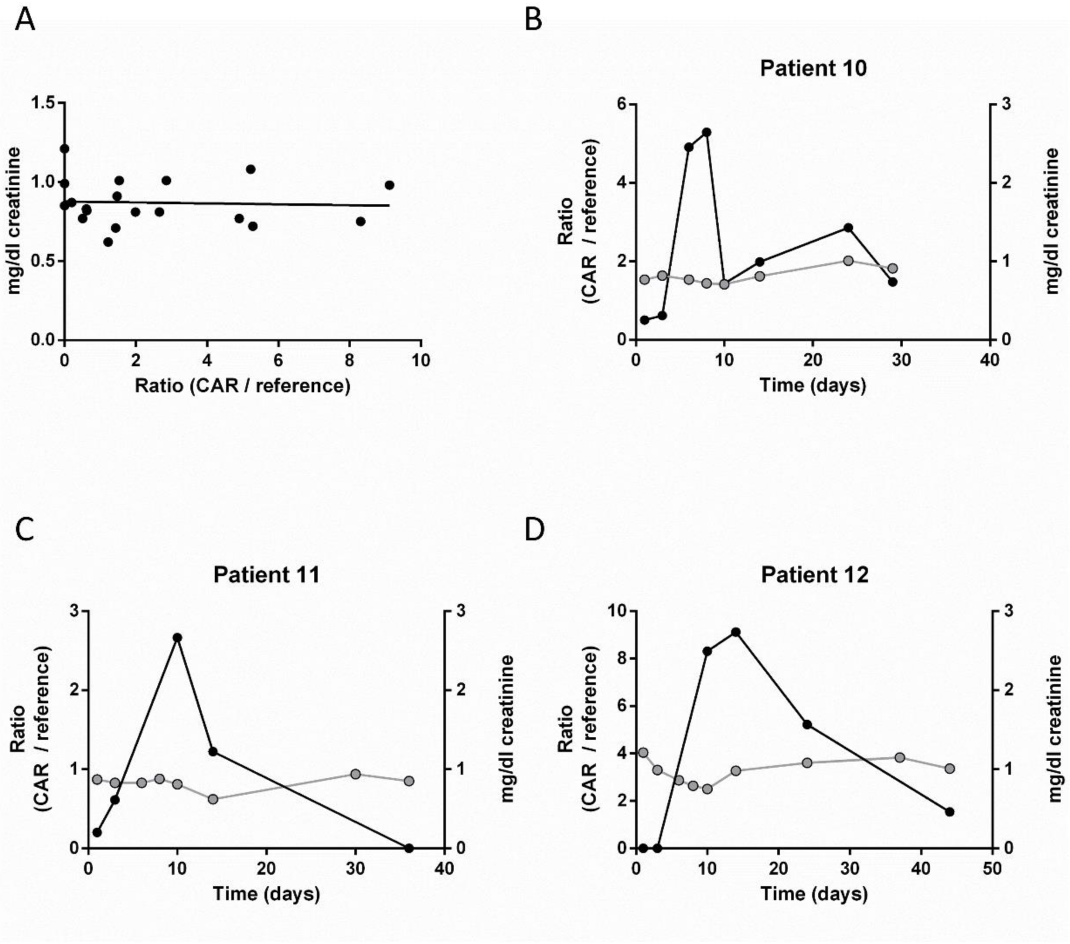

Supplement Figure 8: **Correlation of cfDNA and renal function**

A: Pearson correlation of serum creatinine levels and the ratio of the cfCAR-DNA ($r^2 = 0.003$).

B-D: The ratio of cfCAR-DNA to reference cfDNA and serum creatinine levels of patients treated with axi-cel over time. Black dots: ratio, grey dots: mg/dl creatinine.

Supp. Fig. 9

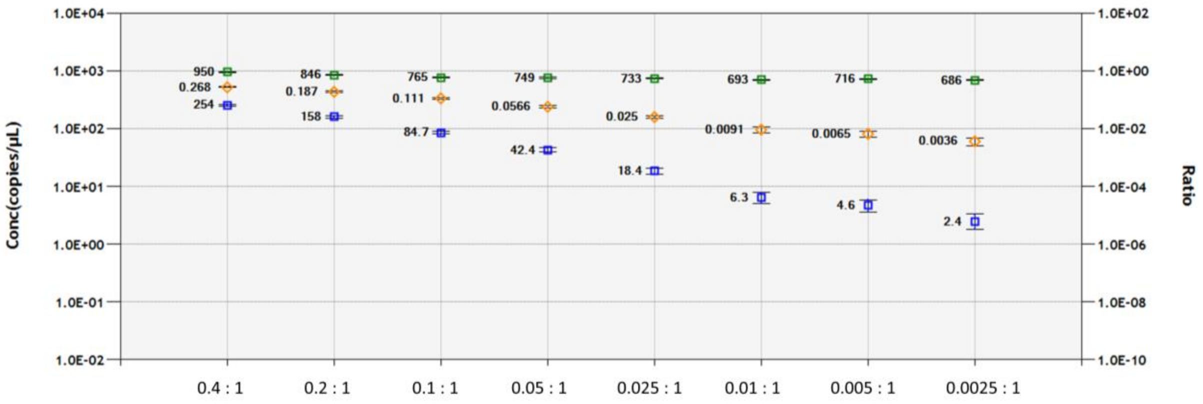

***Supplement Figure 9*:** **Dilution of Karpas derived gDNA in reference gDNA**

Karpas gDNA, comprising IgH-BCL2 translocation, was spiked into 50 ng of reference human gDNA. Subsequently, IgH-BCL2 copies were assessed by an assay previously developed by Drandi et al. [37]. IgH-BCL2 copies were reliably detectable in a ratio of 0.0025 (2.5 x 10^{-3}).
